# Actomyosin contractility confers mechanoprotection against TNFα-induced disruption of the intervertebral disc

**DOI:** 10.1126/sciadv.aba2368

**Published:** 2020-08-19

**Authors:** Paula A. Hernandez, Timothy D. Jacobsen, Nadeen O. Chahine

**Affiliations:** 1 Department of Orthopaedic Surgery, University of Texas Southwestern Medical Center, Dallas, TX, USA.; 2 Department of Biomedical Engineering, Columbia University, New York, NY, USA.; 3 Department of Orthopedic Surgery, Columbia University, New York, NY, USA.

## Abstract

Inflammation triggers degradation of intervertebral disc extracellular matrix (ECM), a hallmark of disc degeneration that contributes to back pain. Mechanosensitive nucleus pulposus cells are responsible for ECM production, yet the impact of a proinflammatory microenvironment on cell mechanobiology is unknown. Using gain- and loss-of-function approaches, we show that tumor necrosis factor–α (TNFα)–induced inflammation alters cell morphology and biophysical properties (circularity, contractility, cell stiffness, and hydraulic permeability) in a mechanism dependent on actomyosin contractility in a three-dimensional (3D) culture. We found that RhoA activation rescued cells from TNFα-induced mechanobiological disruption. Using a novel explant-in-hydrogel culture system, we demonstrate that nuclear factor kappa-B nuclear translocation and transcription are mechanosensitive, and its downstream effects on ECM degradation are regulated by actomyosin contractility. Results define a scaling relationship between circularity, contractility, and hydraulic permeability that is conserved from healthy to inflammatory microenvironments and is indicative of cell mechanobiological control across scales in 3D.

## Teaser

Rho Rho Rho your cells: Increased RhoA activity protects intervertebral disc from TNFα-induced degradation.

## INTRODUCTION

Soft tissue degradation is a hallmark of structural diseases, such as degenerative intervertebral disc (IVD) disease. Low back pain, a major cause of disability worldwide, is associated with disc degeneration (DD) in as many as 40% of cases (*1*). Degeneration is characterized by changes in cellular and biochemical homeostasis leading to loss of extracellular matrix (ECM) proteoglycans (PGs), increased fibrillation, and lower water content (*2*), compromising both the structural integrity and function of the IVD. Proinflammatory cytokine expression, namely, tumor necrosis factor–α (TNFα), interleukin-1β (IL-1β), and IL-6, and release of nitric oxide (NO) increase with severity of DD in patients (*3*–*5*). In vivo studies have shown that inflammatory stimulation of the IVD with TNFα or lipopolysaccharide is sufficient to trigger DD, loss of disc height, and matrix breakdown (*6*) and induces pain behavior in rats (*7*). Ex vivo explant cultures showed that TNFα stimulation decreased ECM and increased catabolic gene expression, along with loss of explant tissue integrity and GAG (Glycosaminoglycan) content (*8*–*10*). In agreement, two-dimensional (2D) cell culture studies confirm that TNFα and IL-1β alter disc cell phenotype, favoring up-regulation of catabolic mediators, suppression of ECM genes, and induction of cell senescence and autophagy (*4*, *8*, *10*, *11*).

Tissues and cells are exposed to a variety of mechanical environments in vivo. As part of the physiological functioning of the disc, nucleus pulposus (NP) cells are exposed to mechanical, hydrostatic, and osmotic loads (*12*). Osmotic and hydrostatic signals are of key importance in the IVD due to its highly hydrated nature. Diurnal changes in IVD hydration, as part of the normal functioning of the disc and in response to degeneration and GAG loss, lead to changing osmotic and hydrostatic signals experienced by cells. Cells are mechanosensitive and respond to mechanical loading in a number of ways, including changes in cell volume regulation and cytoskeletal organization, which lead to altered gene expression and protein synthesis (*13*, *14*). Furthermore, in response to damaging mechanical stimuli such as hyperphysiologic loading, disc cells have a catabolic response, including loss of ECM synthesis and increased production of matrix metalloproteinases (MMPs) (*15*, *16*) similar to the response induced by inflammatory stimulation. Therefore, there is a preponderance of evidence that ECM loss due to inflammatory stimuli or mechanical overloading shares common pathways. However, there is a lack of direct evidence of cellular and molecular cross-talk between mechanobiological and inflammatory signaling in NP cells.

Addressing the gap between mechanobiology and inflammation, we have previously shown that proinflammatory stimulation of single cells leads to significant changes in biomechanical properties, including increased cell hydraulic permeability and increased cell size. In addition, we have shown that this inflammatory stimulation leads to disruption of the actin cytoskeleton (*17*). Changes in cell morphology, including the extension of cytoplasmic processes, also occur under pathological conditions of the disc (*18*, *19*), yet the driving forces for these morphological changes are unknown. Actin cytoskeleton and actomyosin contractility are regulators of cell shape, where a balance of contractile forces drives shape changes, such as cell spreading or round morphology. These forces are controlled by phosphorylation of the regulatory myosin light chain (MLC) and myosin heavy chains (*20*). The role of actomyosin contractility as a mechanism by which TNFα induces changes in NP cell biomechanical properties, cell morphology, and cytoskeletal organization is unknown. We hypothesize that TNFα-induced changes in cell biophysical properties are mediated by a loss of actomyosin contractility; therefore, increasing actomyosin contractility can prevent TNFα-induced alterations to cell biophysical properties. Using gain- and loss-of-function experimental approaches, we show that the proinflammatory cytokine TNFα disrupts cell biomechanical properties (hydraulic permeability and elastic modulus) in a manner dependent on Rho guanosine triphosphatase (GTPase) regulation of actomyosin contractility. Furthermore, we find that actomyosin contractility regulates the master proinflammatory transcription factor nuclear factor κB (NF-κB), conferring mechanoprotection against the degradative effects of TNFα at the matrix level. Using 3D cell and explant-in-hydrogel culture models, this study reveals that TNFα-induced tissue degradation is regulated by RhoA-mediated decrease in actomyosin contractility.

## RESULTS

### TNFα alters NP cells morphology and cortical contractility

**Fig. 1:**
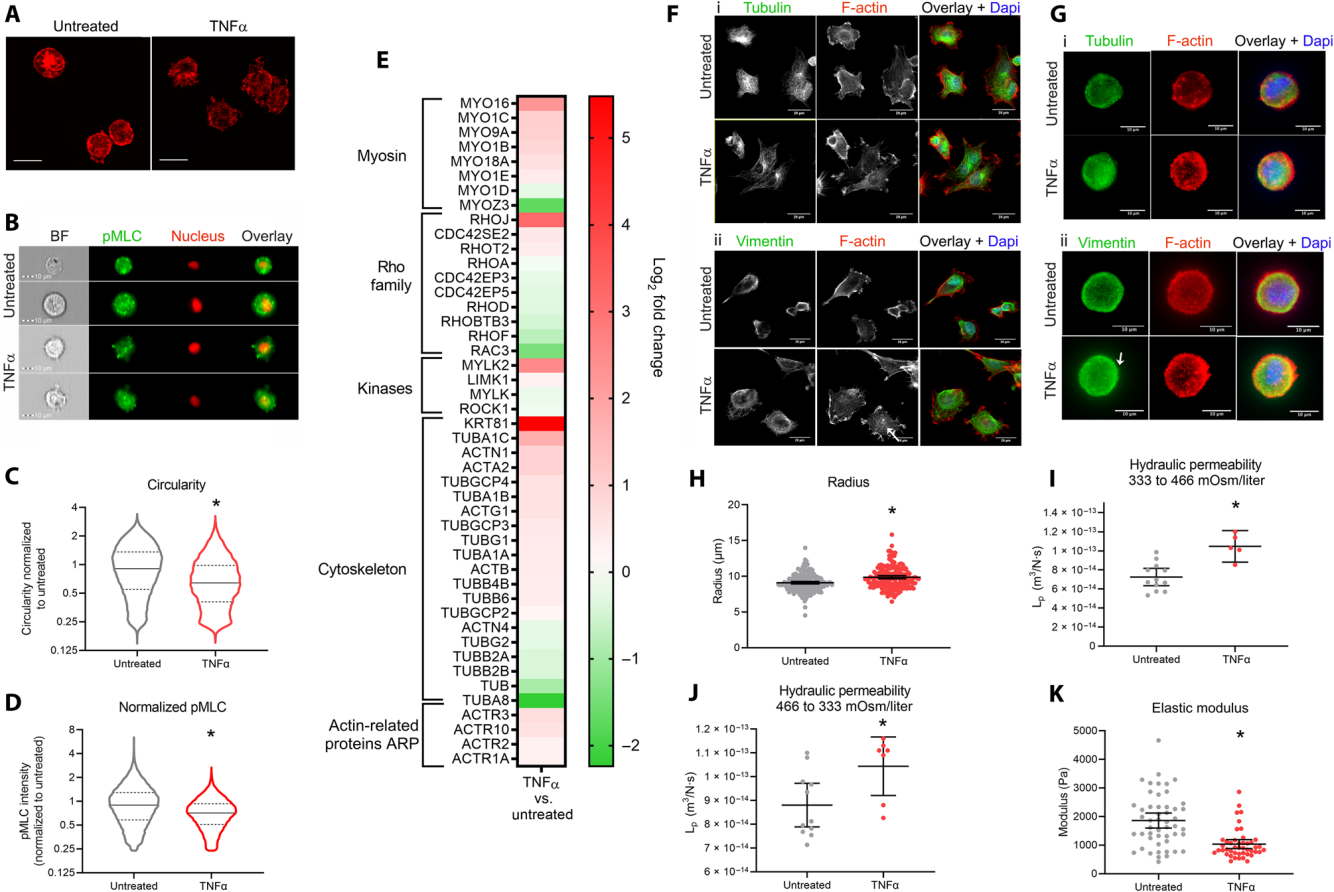
Stimulation of NP cells with TNFα alters cellular morphological and biophysical properties. (**A**) NP cells in beads maintained round morphology. TNFα increased F-actin–rich processes. Scale bars, 20 μm. (**B**) Untreated and TNFα-treated NP cells from imaging flow cytometry [brightfield (BF), pMLC (green), nucleus (red)]. Scale bars, 10 μm. (**C**) Violin plots of circularity and (**D**) pMLC intensity demonstrate that TNFα decreased both (*P < 10^−7^ versus untreated). Solid lines, median; dotted lines, quartiles. (**E**) Heatmap (log_2_ fold change) of significantly changed myosin, Rho GTPases, cytoskeleton, and ARP (Actin-Related Proteins) genes in TNFα versus untreated groups. (**F**) TNFα-treated NP cells grown in 2D have more peripheral F-actin (red). No differences in microtubules were observed (i) (tubulin, green). Vimentin (ii) (green) appeared more peripherally spread in TNFα (white arrow. Scale bars, 20 μm. DAPI, 4′,6-diamidino-2-phenylindole. (**G**) NP cells in alginate exhibited similar microtubules in untreated versus TNFα. Peripheral vimentin intensity increased in TNFα, with small vimentin-rich processes observed (white arrow). Scale bars, 10 μm. TNFα treatment decreased (**H**) cell radius (*P < 10^−7^ versus untreated, *n* = 180 to 193) and increased (**I**) hyperosmotic *L*_p_ (*P < 10^−4^ versus untreated, *n* = 5 to 12) and (**J**) hypo-osmotic *L*_p_ (*P = 0.024 versus untreated, *n* = 7 to 11). (**K**) Cell modulus decreased with TNFα treatment (*P < 10^−7^ versus untreated, *n* = 45 to 50). (H) to (K) represent means ± SD.

In our prior study, we observed that cells treated with TNFα in 2D had a higher hydraulic permeability compared to untreated cells (*17*). To assess the effect of TNFα stimulation on cell biomechanics and cytoskeletal structure in a more physiologic 3D model, we cultured juvenile bovine NP cells in alginate beads to more closely mimic their native cell shape. Under these conditions, cells maintain a rounded morphology; however, in the same environment, when exposed to TNFα (10 ng/ml), they developed more F-actin–rich extensions (Fig. 1A). To quantify changes in cell morphology, we imaged and quantitatively analyzed cells in a high-throughput manner using imaging flow cytometry (Amnis ImageStream) (Fig. 1B). The addition of TNFα significantly decreased cell circularity (Fig. 1C) from a near-perfect circle under untreated conditions [mean (95% CI), 1.0 (0.995 to 1.00)] to a more irregular morphology in TNFα [0.746 (0.739 to 1.754), *P* < 10^−7^]. We also evaluated actomyosin contractility changes by quantifying phosphorylated MLC (pMLC) using imaging flow cytometry (Fig. 1, B and D). Because of rapid turnover of MLC phosphorylation, cells were fixed and stained immediately at the end of inflammatory stimulation while cells were inside the alginate beads, therefore preventing any disturbance provoked by the release of cells from beads for analysis. No major differences were observed in morphology or cell extensions in TNFα-treated cells imaged within or released from the bead (fig. S1). Levels of pMLC significantly decreased [0.804 (0.799 to 0.808)] in TNFα treatment compared to untreated [1.0 (0.996 to 1.004), *P* < 10^−7^] (Fig. 1D). We examined contractility transcriptome in NP cells using RNA sequencing (RNA-seq) and found that expression of Rho GTPases RhoA, RhoD, RhoF, and CDC42 significantly decreased in TNFα-treated cells, while expression of LIMK1, RhoJ, and RhoT2 significantly increased (Fig. 1E). Analysis with Reactome further demonstrated that TNFα treatment decreased expression of genes in Rho GTPase signaling pathway including guanine nucleotide exchange factors and GTPase-activating proteins (GAPs) and MLC kinase (MYLK). Expression of Rho-associated protein kinase-1 (ROCK-1) and Rho GTPase effector citron kinase-3 (CIT3) also decreased. Expression of additional targets shared by ROCK and CIT pathways including nonmuscle myosin II genes [myosin light polypeptide-6 (MYL6), MYL9, and MYH9 (Myosin heavy chain-9)] significantly decreased (fig. S2). We also investigated the effects of TNFα on tubulin and vimentin staining in 2D and in 3D (Fig. 1, F and G). In 2D and 3D, cellular extensions were enriched with vimentin intermediate filaments; however, no changes in tubulin were observed (Fig. 1, F and G). Since cell morphology can be affected by serum levels in the tissue culture media [e.g., serum stimulation favors cell growth, while serum starvation enhances stress fiber formation and fibroblastic phenotype (*21*)] and to promote rounded cell morphology in our 3D model, cells were grown in the presence of 10% serum. Nevertheless, experiments performed with low 1% serum showed similar effects of TNFα when compared to a respective untreated group cultured in 1% serum, although observations of cell extensions rich in F-actin and vimentin were more prevalent in all groups cultured in 1% compared to 10% serum (fig. S1).

### Effect of TNFα on hydraulic permeability and cell stiffness

To assess the impact of morphological and actomyosin contractility changes on cell biophysical properties, we measured changes in cell hydraulic permeability and stiffness after stimulation with TNFα in 3D alginate culture. The cell radius under isotonic conditions (333 mOsm/liter) significantly increased in TNFα-treated cells compared to untreated cells (means ± SD; untreated: 9.1 ± 1.3 μm, TNFα: 9.9 ± 1.5 μm, *P* < 0.001; Fig. 1H). Since changes in cell volume can be related to regulation of osmotic pressure, we measured the hydraulic permeability (*L*_p_) under two osmotic environments. Cells were seeded into a microfluidic channel and were exposed to a hyperosmotic step load (466 mOsm/liter) to induce cell shrinking, followed by a hypo-osmotic step load (333 mOsm/liter) to induce cell swelling. Cell volume changes were tracked in real time using differential interference contrast (DIC) imaging and a microfluidic channel (*22*, *23*). Volume change over time was curve-fitted using a mixture theory model (*17*, *22*, *23*) to derive *L*_p_ of NP cells from TNFα-treated and untreated groups under hypo- and hyperosmotic conditions. During hyperosmotic load (333 to 466 mOsm/liter), cells treated with TNFα had a significant increase in *L*_p_ (10.5 ± 1.3 × 10^−14^ m^3^/N·s, means ± SD) compared to untreated cells (7.2 ± 1.4 × 10^−14^ m^3^/N·s, *P* < 0.001), indicating that TNFα-treated cells were more permeable to water flow out of the cell during cell shrinking (Fig. 1I). When cells were exposed to a hypo-osmotic load (466 to 333 mOsm/liter), *L*_p_ in TNFα group was also elevated (10.4 ± 1.3 × 10^−14^ m^3^/N·s) compared to untreated group (8.8 ± 1.4 × 10^−14^ m^3^/N·s, *P* < 0.05; Fig. 1J). The osmotically active water content (F_*ir*_) for TNFα-treated cells was significantly lower for cells exposed to hypo-osmotic stress but did not change for cells exposed to hyperosmotic stress (fig. S3) (*17*). To investigate mechanical changes of cells under compression (representative of the compressive environment of the IVD), we performed microindentation testing of cells with atomic force microscopy (AFM) to obtain force-indentation curves, which were used to calculate cell stiffness under normal and TNFα conditions. Cells treated with TNFα had significantly lower elastic modulus (1.04 ± 0.52 kPa, means ± SD) compared to untreated cells (1.86 ± 0.91 kPa, *P* < 10^−5^) (Fig. 1K).

### Inhibiting myosin-II contractility mimics the effect of TNFα on cell morphology and biophysical properties

**Fig. 2:**
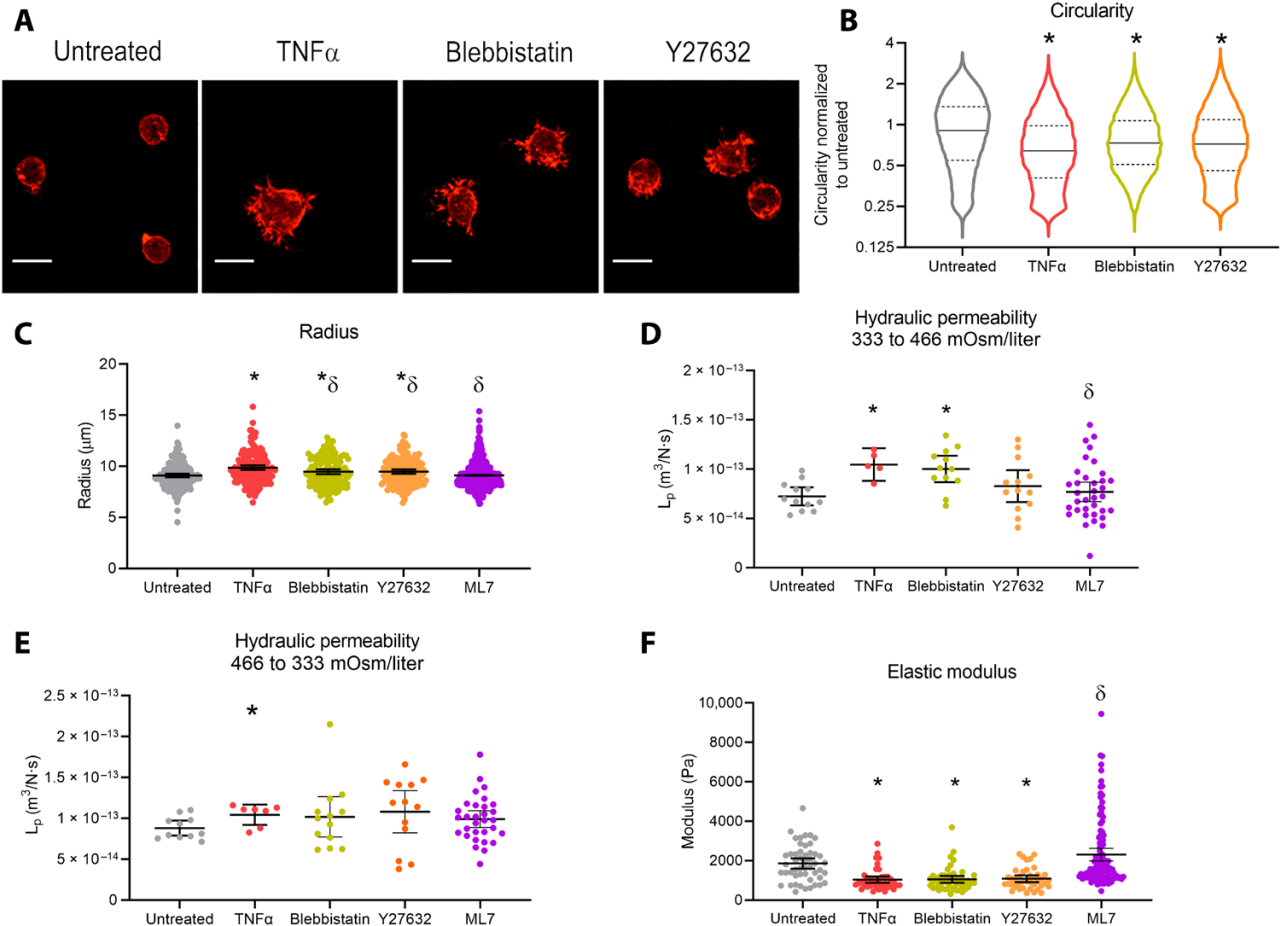
Inhibiting myosin-II mimics the effects of TNFα on NP cell morphology and single-cell biophysical properties. (**A**) F-actin–rich processes were observed in NP cells treated with TNFα (10 ng/ml), 10 μM blebbistatin, and 10 μM Y27632 for 24 hours compared to untreated cells. Scale bars, 20 μm. (**B**) Circularity significantly decreased in all treatments (*P < 10^−7^ versus untreated). (**C**) Cell radius increased in blebbistatin and Y27632 groups, similar to TNFα (*P < 0.02 versus untreated, *n* = 133 to 193) but not in ML7. δ indicates *P* < 0.05 compared to TNFα. (**D**) *L*_p_ during hyperosmotic loading significantly increased in blebbistatin and TNFα groups but not in Y27632 or ML7 (*P < 0.01 versus untreated, *n* = 5 to 13). δ indicates *P* < 0.05 compared to TNFα. (**E**) *L*_p_ during hypo-osmotic loading was significantly higher in the TNFα group but not in blebbistatin, Y27632, or ML7 (*P < 0.02 versus untreated, *n* = 7 to 13). (**F**) Single-cell elastic modulus significantly decreased in TNFα, Y27632, and blebbistatin but not in ML7 (*P < 10^−6^ versus untreated, *n* = 40 to 50). δ indicates *P* < 0.05 compared to TNFα. Scatter plot lines in (C) to (F) represent means ± SD.

To evaluate the potential contributions of cortical contractility to hydraulic permeability, we modulated contractility by treating NP cells with drugs affecting specific contractility mechanisms. NP cells were treated with blebbistatin, a potent myosin-II inhibitor, Y27632, a ROCK inhibitor, or ML7, a MYLK inhibitor. Actin fiber staining after treatments showed that both blebbistatin and Y27632 induced extension of cell processes for cells in 3D, consistent with that seen in TNFα treatment (Fig. 2A). Measurements of cell circularity showed a significant decrease in blebbistatin- [0.839 (0.832 to 0.846), *P* < 10^−7^] and Y27632-treated cells [0.836 (0.828 to 0.843), *P* < 10^−7^; Fig. 2B] compared to untreated NP cells, confirming that preventing actomyosin cross-linking results in loss of rounded cell morphology and the formation of cell processes in a 3D cell culture system. Cell radius measured under isotonic conditions revealed that both blebbistatin and Y27632 mimic the effects of TNFα treatment with an increased cell radius (untreated: 9.1 ± 1.3 μm, blebbistatin: 9.5 ± 1.4 μm, *P* < 0.02 versus untreated; Y27632: 9.5 ± 1.3 μm, *P* < 0.02 versus untreated), while radius of ML7-treated cells was comparable to that of the untreated cells (9.12 ± 1.3 μm; Fig. 2C). To further analyze the effect of disrupting specific contractility mechanisms on hydraulic permeability and cell stiffness, we found that blebbistatin increased the *L*_p_ during hyperosmotic loading from 7.2 ± 1.4 × 10^−14^ m^3^/N·s in untreated to 10.0 ± 2.1 × 10^−14^ m^3^/N·s (*P* < 0.03), similar to what was observed with TNFα. Neither Y27632 nor ML7 produced a significant effect on *L*_p_ (Y27632: 8.3 ± 2.7 × 10^−14^ m^3^/N·s, ML7: 7.7 ± 2.9 × 10^−14^ m^3^/N·s; Fig. 2D). When cells were exposed to hypo-osmotic loading, none of the treatments showed significant effect on *L*_p_ compared to the untreated group (Fig. 2E). The osmotically active water content (F*_ir_*) for both osmotic loading steps showed no significant difference with inhibitor treatments (fig. S3). In evaluating cell stiffness, blebbistatin and Y27632 significantly decreased elastic modulus compared to untreated, while ML7 had no significant effect (untreated: 1.86 ± 0.91 kPa, blebbistatin: 1.05 ± 0.51 kPa, *P* < 10^−5^; Y27632: 1.09 ± 0.53 kPa, *P* < 10^−7^; ML7: 2.25 ± 1.72 kPa; Fig. 2F). The modulus in blebbistatin and Y27632 groups was comparable to values observed in the TNFα-treated group (1.04 ± 0.52 kPa). These findings show that inhibiting myosin-II most closely mimics the effects of TNFα on NP cell morphology, contractility, stiffness, and hyperosmotic permeability. Inhibiting ROCK resulted in partial phenocopying, whereas inhibiting MYLK had no major effect. To determine whether contractility inhibitors had additional effects beyond that of TNFα, we examined the potential for additive effects of TNFα with blebbistatin or Y27632. No further decreases in cell circularity were observed in cells cotreated with blebbistatin + TNFα or Y27632 + TNFα (fig. S4). Since Cdc42 expression decreased with TNFα treatment, we also interrogated whether ML141, a Cdc42 inhibitor, phenocopied the effects of TNFα. No significant change in circularity was observed with ML141 treatment. Moreover, cotreatment with ML141 + TNFα had no additional effect on circularity compared to TNFα alone (fig. S5).

### Activating RhoA protects against mechanobiological effects of TNFα

**Fig. 3:**
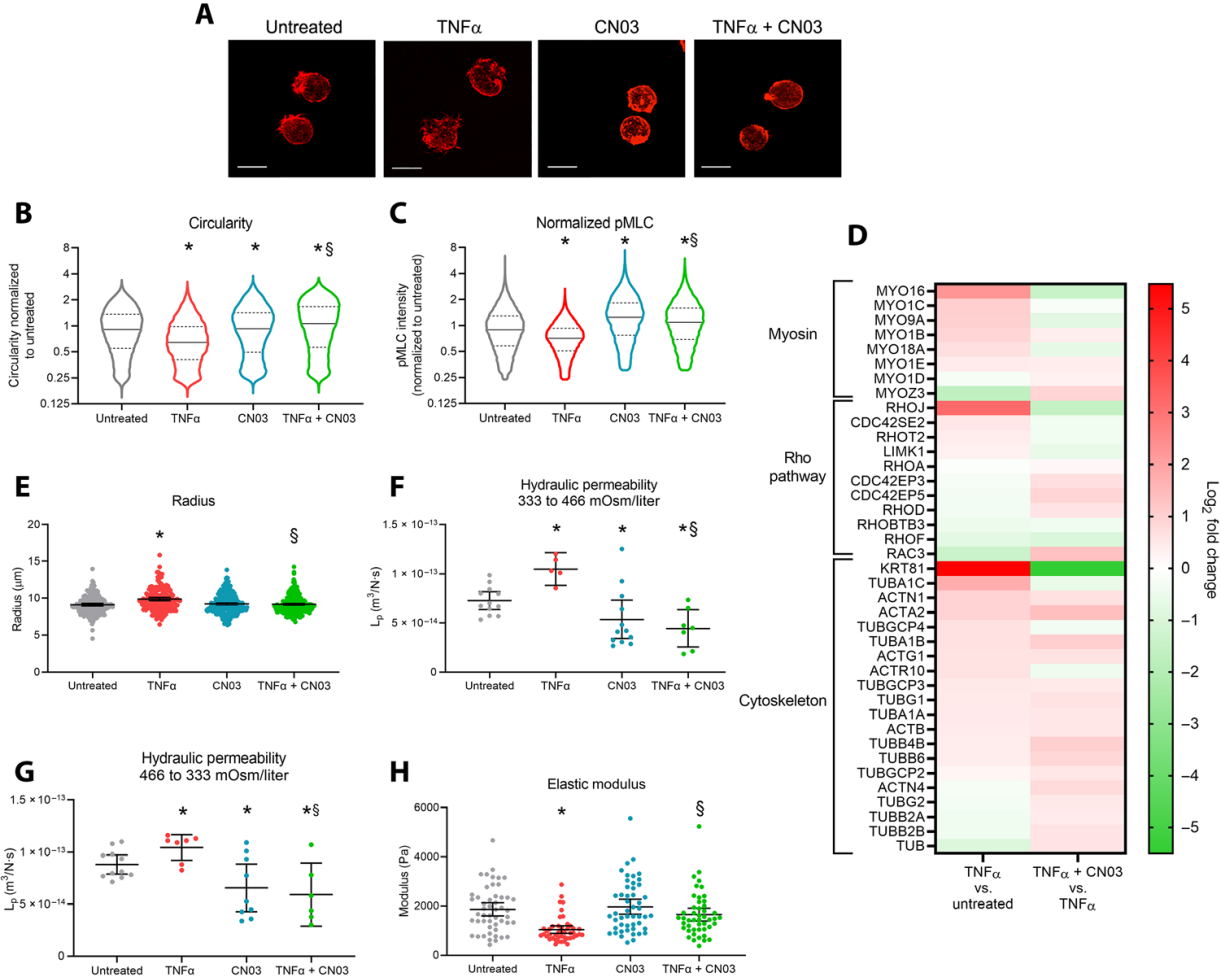
Increasing actomyosin contractility with the RhoA activator CN03 protects against TNFα-induced morphological and biophysical changes. (**A**) NP cells treated with CN03 or CN03 + TNFα have similar morphology to untreated cells (F-actin, red). Scale bars, 20 μm. (**B**) CN03 + TNFα increased circularity versus TNFα to a level beyond that of untreated cells (*P < 10^−7^ versus untreated, §*P* < 10^−7^ versus TNFα, *n* = 13,000 to 20,000). (**C**) CN03 + TNFα protected against TNFα-induced decrease in pMLC levels [*n* and *P* values are the same as in (B)]. (**D**) Heatmap (log_2_ fold change) of significantly changed myosin, Rho GTPases, cytoskeleton, and ARP genes in TNFα versus untreated and CN03 + TNFα versus TNFα groups. (**E**) CN03 + TNFα cell radius was significantly decreased versus TNFα (*P < 10^−7^ versus untreated, §*P* < 10^−7^ versus TNFα, *n* = 180 to 239). (**F**) Hyperosmotic *L*_p_ in CN03 + TNFα significantly decreased versus TNFα or untreated (*P < 0.05 versus untreated, §*P* < 0.001 versus TNFα, *n* = 5 to 12). (**G**) Hypo-osmotic *L*_p_ significantly decreased in CN03 + TNFα versus TNFα (*P < 0.03 versus untreated, §*P* < 10^−6^ versus TNFα, *n* = 6 to 11). (**H**) Cell modulus in CN03 + TNFα increased versus TNFα, approaching untreated levels (*P < 10^−6^ versus untreated, §*P* < 0.001 versus TNFα, *n* = 45 to 50). (E) to (H) are means ± SD.

Since inhibition of actomyosin cross-linking mimics the effects of TNFα on cell morphology and biomechanics, we investigated whether the inverse (i.e., increasing actomyosin contractility) can prevent TNFα-induced changes in cell morphology and biomechanical properties. For this, we treated NP cells with CN03, an activator of RhoA, thus increasing actomyosin contractility. CN03 activates Rho GTPase isoforms by blocking intrinsic and GAP-stimulated GTPase activity, resulting in constitutively active Rho (*24*). F-actin staining showed that CN03-treated cells had a marked round morphology compared to untreated cells, confirming that an increase in cortical contractility promotes roundedness and decreases the number of cell extensions (Fig. 3A). Circularity of cells cotreated with CN03 + TNFα [1.167 (1.156 to 1.178)] was greater than that of TNFα-treated cells [0.746 (0.739 to 0.754), *P* < 10^−7^; Fig. 3B). This demonstrates that decreased actomyosin contractility mediates the morphological response of NP cells to TNFα stimulation. Further supporting this hypothesis is the observation that RhoA activation protected against TNFα-induced decrease in pMLC levels, resulting in levels that approached those of the untreated cells [CN03 + TNFα: 1.123 (1.116 to 1.131) versus TNFα: 0.804 (0.799 to 0.808), *P* < 10^−7^; Fig. 3C). CN03 cotreatment also prevented the increase in cell radius induced by TNFα, returning levels to that measured in the untreated group (CN03 + TNFα: 9.5 ± 0.08 μm, TNFα: 9.9 ± 0.1 μm, untreated: 9.1 ± 0.09 μm, *P* < 10^−7^ for CN03 + TNFα versus TNFα, *P* = 0.7 for CN03 + TNFα versus untreated), further demonstrating the protective effects of Rho pathway activation on TNFα-induced cellular contractility (Fig. 3D). Transcriptome analysis demonstrated that CN03 + TNFα exhibited significant increases in Rho GTPase and cytoskeleton expression compared to TNFα (Fig. 3H). Reactome pathway analyses revealed significant increases in expression of CIT, formin, and Ras IQGAP (Ras GTPase-activating–like protein) pathways but not in ROCK pathways. Expression of RhoA, RhoC, RhoD, and CDC42 GTPases increased, along with formin pathway effectors Diaphanous Related Formin 3 (DIAPH3) and Formin-like 3 (FMNL3). CIT pathway effector CIT3 significantly increased along with expression of myosin-II–related genes MYL6, MYL9, MYH9, and MYH10 in CN03 + TNFα compared to TNFα (fig. S6). Next, we investigated whether RhoA activation also prevents biophysical changes induced by TNFα. Under hyperosmotic loading, RhoA activation (4.4 ± 2.0 × 10^−14^ m^3^/N·s) rescued the increase in *L*_p_ observed in TNFα alone (10.5 ± 1.3 × 10^−14^ m^3^/N·s, *P* < 0.001; Fig. 3E). Similar effects were observed for hypo-osmotic *L*_p_ (CN03 + TNFα: 5.9 ± 2.9 × 10^−14^ m^3^/N·s, TNFα: 10.4 ± 1.3 × 10^−14^ m^3^/N·s, *P* < 10^−4^; Fig. 3F). CN03 + TNFα had no appreciable effect on osmotically active water content compared to TNFα treatment in either osmotic step (fig. S3). Cell stiffness measurements by AFM revealed that the decreases in elastic modulus with TNFα stimulation (1.04 ± 0.52 kPa) were restored to untreated levels (1.86 ± 0.91 kPa) when cells were cotreated with CN03 + TNFα (1.65 ± 0.88 kPa, *P* = 0.544 versus untreated, *P* < 10^−3^ versus TNFα; Fig. 3G). These findings demonstrate that increased RhoA contractility can sufficiently control TNFα-induced biophysical changes of NP cells in 3D.

### Increasing actomyosin contractility blocked NF-κB translocation and inhibited transcription

**Fig. 4:**
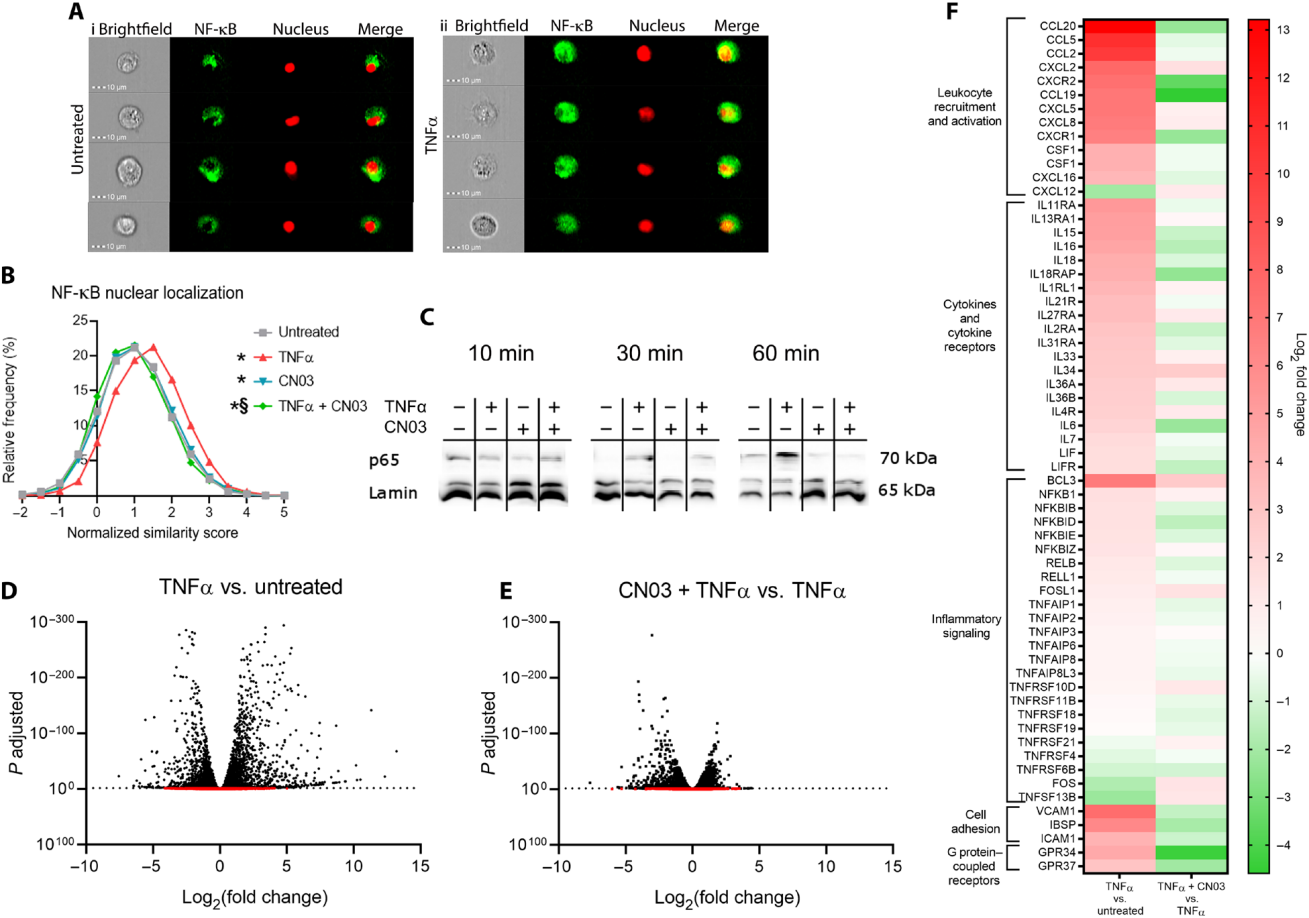
Increasing actomyosin contractility decreases NF-κB nuclear localization and alters expression levels of TNFα-responsive genes. (**A**) Representative images of NP cells from untreated and TNFα-treated groups using imaging flow cytometry, showing brightfield, NF-κB (green), nucleus (Draq5, red), and merged NF-κB + Draq5 images. Scale bars, 10 μm. (**B**) Histograms of NF-κB nuclear colocalization (similarity score calculated by the Amnis IDEAS software) and normalized to untreated group showed an increase with TNFα stimulation that was mitigated with CN03 cotreatment (*P < 10^−5^ versus untreated, §*P* < 10^−7^ versus TNFα). (**C**) Western blot of nuclear fraction of NP cells from TNFα ± CN03 treatment groups. Cell lysates were isolated after 10, 30, or 60 min of treatment, showing NF-κB (p65) and loading control for nuclear fraction (lamin). Inflammatory stimulation led to increased nuclear NF-κB at all time points. Cotreatment with CN03 + TNFα decreased p65 at 30 and 60 min. (**D**) Volcano plot of transcriptomics data obtained for TNFα versus untreated. (**E**) Volcano plot of transcriptomics for CN03 + TNFα versus TNFα. Dotted line indicates threshold of significance *P* = 0.05. In red are genes with nonsignificant changes, and in black are genes with significant changes. (**F**) Heatmap (log_2_ fold change) of TNFα-responsive genes and NF-κB signaling genes that were found to have significant change [*P*_adj_ (adjusted *P* value) < 0.05] in TNFα versus untreated and CN03 + TNFα versus TNFα groups (color bar, log_2_ fold change).

NF-κB is a master transcription factor regulating proinflammatory responses in NP cells (*25*). In response to specific signals, the NF-κB dimer translocates from its resting state in the cytoplasm to the nucleus where it regulates the transcription of genes; therefore, nuclear localization is indicative of NF-κB activation. There is some small evidence that NF-κB can act as a mediator of mechanotransduction (e.g., in osteoblasts) (*26*). The role of NF-κB in mechanotransduction of NP cells is largely unknown, including the effects of actomyosin contractility on the regulation of NF-κB activity. Therefore, we investigated how levels and nuclear localization of NF-κB in 3D cultured NP cells varied with altered actomyosin contractility by comparing cells treated with TNFα, CN03, or CN03 + TNFα. We evaluated these changes by performing high-throughput image analysis of NF-κB nuclear colocalization of NP cells in 3D culture using imaging flow cytometry (*27*). Imaging flow cytometry analysis showed that TNFα stimulation of NP cells significantly increased nuclear NF-κB colocalization compared to untreated cells [TNFα: 1.377 (1.353 to 1.402), untreated: 1.0 (0.976 to 1.024), *P* < 10^−7^; Fig. 4, A and B], while RhoA activation reduced NF-κB nuclear localization to levels similar to those in the untreated group [CN03 + TNFα: 0.986, (0.961 to 1.010), *P* < 10^−7^ versus TNFα; Fig. 4B]. Further validating the imaging flow cytometry results, Western blot analysis (Fig. 4C) showed that TNFα increased the nuclear p65 NF-κB subunit at 10, 30, and 60 min of stimulation, indicative of NF-κB activation. At 30 and 60 min, CN03 + TNFα cotreatment led to decreased nuclear p65 compared to TNFα alone. To inform the biological relevance, volcano plots show significant changes due to TNFα versus untreated and CN03 + TNFα versus TNFα (Fig. 4, D and E). Transcriptome analyses indicate increased expression of genes downstream of NF-κB based on the Kyoto Encyclopedia of Genes and Genomes (KEGG) pathway definition, including significant increases in cytokines, chemokines, and NF-κB signaling pathway in TNFα versus untreated groups (Fig. 4F). Reactome analyses indicate that IL-1 family signaling pathway significantly increased and noncanonical NF-κB pathways mediated by NF-κB–inducing kinase (*NIK*), *TNF receptor 2*, and *Dectin-1* are all up-regulated with TNFα treatment (fig. S7). Cotreatment with CN03 + TNFα had associated decreases in subsets of inflammatory-related genes, including cytokines [e.g., *IL-6*, *IL-7*, *leukemia inhibitory factor* (*LIF*), and *LIF* *receptor*], chemokines (e.g., *CCL2*, *CCL5*, *CCL19*, *CCL20*, *CXCR1*, and *CXCR2*), and NF-κB signaling (*NFKB1B*, *NFKB1D*, *NFKB1E*, *RELB*, and *RELL1*; Fig. 4F). Similarly, cotreatment resulted in significant inhibition of IL-1 signaling pathway, NF-κB activation pathway, and NIK noncanonical NF-κB signaling pathway. These results demonstrate that in the presence of an inflammatory stimulus, activation of RhoA can block NF-κB nuclear translocation and alter transcriptional levels of canonical and noncanonical signaling pathways. These findings demonstrate that increased actomyosin contractility mediates TNFα-induced NF-κB activity, establishing a cross-talk between inflammatory signaling and cellular mechanobiology in a connective tissue cell type.

### Increasing actomyosin contractility protects against cell morphological change and GAG loss in NP tissue

**Fig. 5:**
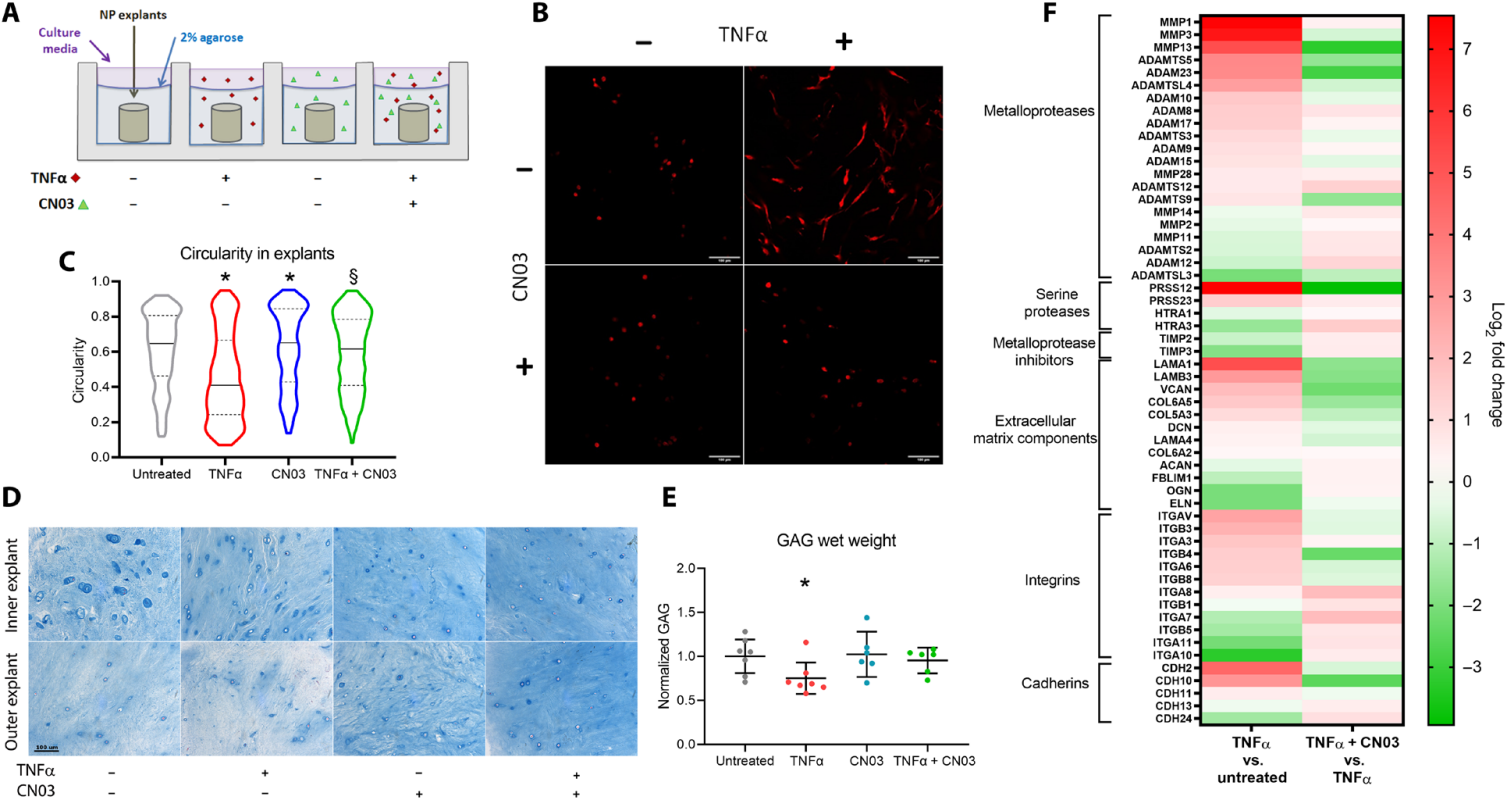
Increasing actomyosin contractility protects tissue from TNFα-induced GAG loss. (**A**) Schematic of the explant-in-hydrogel culture system and respective treatment groups. NP explants were cast and cultured within 2% agarose hydrogel to prevent swelling and treated with culture media only (untreated), TNFα, CN03, or CN03 + TNFα for 14 days. (**B**) Confocal fluorescence of NP explant sections stained with phalloidin for F-actin visualization. Cells within tissue show that the morphological changes induced by TNFα treatment are prevented by coincubation with CN03. Scale bars, 100 μm. (**C**) Violin plot for circularity of NP cells within explants indicates that the decrease induced by TNFα is mitigated in CN03 + TNFα. Solid lines represent median, and dotted lines represent quartiles. *P < 0.05 compared to untreated, §*P* < 0.05 compared to TNFα. (**D**) Histological sections of NP explants after 14 days in the swelling-restricted organ culture system, stained with Alcian blue to show GAG content. Sections were generated from across the explant cross section, showing effects near the central (inner) and periphery (outer) of the explant. There was a reduction in GAG staining in the outer area with TNFα. Cotreatment with CN03 + TNFα mitigated this GAG loss. Scale bar, 100 μm. (**E**) Quantification of GAG content (percentage of wet weight) measured by DMMB assay of explant digests, normalized to untreated control (means ± SD). Inflammatory treatment with TNFα reduced overall GAG content (*P = 0.04 versus untreated, *n* = 6 to 7). Scatter plot lines in (F) show means ± SD. (**F**) Heatmap (log_2_ fold change) of ECM genes and degradative/catabolic genes that were found to have significant change (*P*_adj_ < 0.05) in TNFα versus untreated and CN03 + TNFα versus TNFα groups (color bar, log_2_ fold change).

To further assess the effect of 3D culture on morphological response of NP cells to TNFα, we evaluated NP cells surrounded by a native ECM using an organ culture model system. NP explants were cultured within a hydrogel system (explant-in-hydrogel) to prevent tissue swelling and maintain long-term cell viability. We have validated that this culture system prevents cell death and mitigates associated inflammatory response and GAG loss, which occur in free-swelling NP explant culture (Fig. 5A and fig. S8). To investigate the impact of cellular actomyosin contractility changes on tissue matrix integrity, we evaluated changes in cell morphology and circularity using the NP explant-in-hydrogel culture system, where tissues were cultured with TNFα ± CN03 for 14 days (Fig. 5, A and B). In the native 3D ECM environment, TNFα stimulation induced formation of actin-rich cell extensions and polarization, which is further supported by a significant decrease in circularity [untreated: 0.61 (0.58 to 0.65), TNFα: 0.46 (0.43 to 0.49), *P* < 10^−7^; Fig. 5C]. These morphological changes were mitigated by cotreatment with CN03 + TNFα, resulting in a significantly increased circularity compared to TNFα alone [CN03 + TNFα: 0.59 (0.56 to 0.62), *P* < 10^−7^ versus TNFα; Fig. 5C]. No significant difference was observed between untreated explants and those cotreated with CN03 + TNFα (*P* = 0.20 versus untreated). These findings from NP cells within an explant further provide evidence that RhoA activation is sufficient to counteract the effects of TNFα-induced morphological changes. Stimulation of the NP tissue with TNFα can lead to catabolic degradation and PG loss (*8*, *10*). Alcian blue staining of NP explants showed lower GAG intensity in TNFα-treated groups compared to untreated or CN03 cotreated groups (Fig. 5D). Spatially dependent responses to TNFα were observed, with the most pronounced effects observed in the explant periphery, due to low diffusivity of the dense NP ECM. TNFα-stimulated explants had significantly lower GAG content [percentage of wet weight (%ww)] as measured by dimethylmethylene blue (DMMB) assay compared to that of untreated explants (TNFα: 0.75 ± 0.19 %ww, untreated: 1.0 ± 0.21 %ww; *P* < 0.04; Fig. 5E). GAG content of CN03 + TNFα (0.95 ± 0.14 %ww) cotreated explants recovered to untreated levels (*P* = 0.8 versus untreated, *P* = 0.09 versus TNFα). CN03 alone had no significant effect on GAG content relative to untreated group (CN03: 1.02 ± 0.25 %ww). Transcriptomic analysis of ECM degradation genes shows that TNFα treatment significantly increased expression of genes associated with matrix catabolism (Fig. 5F). Specifically, we found significant increases in *MMP1*, *MMP3*, *MMP13*, *ADAMTS4*, and *ADAMTS5*, while *MMP2* expression decreased slightly but significantly with TNFα treatment. In addition, we observed that expression of tissue inhibitor of *MMPs* *2* (*TIMP2*) and *TIMP3*, which are natural inhibitors of MMPs, decreased with TNFα treatment. These catabolic changes were accompanied by decreases in ECM gene expression including aggrecan (*ACAN*), the small leucine-rich PGs osteoglycin (*OGN*) and fibromodulin (*FBLIM1*), and elastin (*ELN*). In the CN03 + TNFα group, we found that expression of both *ADAMTS5* and *MMP13* significantly decreased, while expression of *TIMP2* and *TIMP3* significantly increased compared to the TNFα group. These catabolic changes were accompanied by significant increases in *ACAN*, *OGN*, and *FBLIM1* expression. These findings indicate that expressions of the catabolic enzymes (*ADAMTS5* and *MMP13*), their natural inhibitors of catabolic enzymes (*TIMP2* and *TIMP3*), and PG ECM genes (*ACAN* and *OGN*) in response to TNFα treatment are dependent on actomyosin contractility. In all, these results show that TNFα-induced GAG loss is mediated by decreased NP cell actomyosin contractility and that increasing the contractility with RhoA activation is protective of the ECM, a hallmark of DD.

### Universally conserved scaling relationship between contractility, hydraulic permeability, and circularity

**Fig. 6:**
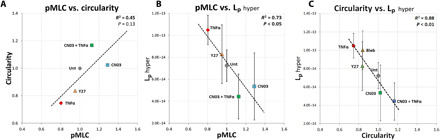
Hydraulic permeability during cell shrinking scales with pMLC levels and actomyosin contractility. (**A**) Trend for a positive correlation between pMLC intensity and cell circularity was observed. Across all treatment groups, we identified a strong inverse correlation between *L*_p_ during hyperosmotic loading and (**B**) pMLC intensity or (**C**) circularity. Linear regression *R*^2^ and *P* values are indicated in each subpanel. In each graph, data points represent the average value from each experimental group (error bars, SD).

Overall, we observed that when cell circularity decreases, i.e., cells develop actin-rich extensions, pMLC levels also decrease, indicative of a loss in cortical contractility. We find a trend for a positive correlation between circularity and pMLC (*R*^2^ = 0.45 and *P* = 0.13; Fig. 6A). We hypothesized and have demonstrated that TNFα impairs the volume regulation of cells by altering actomyosin contractility, which has consequences on *L*_p_. Results indicate that *L*_p_ under hyperosmotic loading and pMLC are inversely correlated (*R*^2^ = 0.73, *P* < 0.05; Fig. 6B). This inverse relationship also holds between *L*_p_ under hyperosmotic loading and circularity (*R*^2^ = 0.88, *P* < 0.01; Fig. 6C). These findings point to a universal scaling relationship between circularity, contractility, and *L*_p_, which is conserved across many microenvironmental conditions, including proinflammatory stimulations, contractility inhibition, and activation. When cells exhibit varying degrees of alterations in pMLC or circularity, a corollary change in hyperosmotic permeability occurs.

## DISCUSSION

This work shows a previously undescribed cell mechanobiology in an inflammatory 3D microenvironment, whereby actomyosin contractility regulates TNFα-induced catabolic expression and NP ECM loss. We further identified NF-κB activity as a mechanism by which increased cortical actomyosin contractility confers mechanoprotection on ECM integrity. Cell contractility mediates nuclear localization of the master transcription factor NF-κB and its downstream catabolic effects on the gelatinous ECM of the NP in a similar mechanism to its regulation of cell morphology and biomechanics. Our data demonstrate that TNFα significantly reduced expression of RhoA, associated Rho GTPase effectors, myosin-II genes, and phosphorylation of MLC, resulting in a direct effect on biophysical properties (decreased elastic modulus and increased hydraulic permeability). Loss-of-function experiments confirmed that decreasing actomyosin contractility with myosin-II inhibitor (blebbistatin) mimics the effects of TNFα on NP cell circularity, permeability, and stiffness. Conversely, gain-of-function experiments showed that increasing actomyosin contractility with RhoA activator (CN03) counteracted the effects of TNFα on cell morphology and biomechanical function. Moreover, we observed that biophysical disruptions due to TNFα are not merely coincidental with the biological effects of TNFα but rather are key mediators of its catabolic effects on cells and tissues. We show that increasing actomyosin contractility prevented TNFα-induced NF-κB translocation, inhibited expression of TNFα responsive genes, and blocked the degradative effect of TNFα on GAG loss from the ECM. These results demonstrate that in a 3D model, cortical actomyosin contractility is sufficient at regulating NF-κB nuclear translocation and controlling its pleiotropic effects on cells and tissues (e.g., catabolic and ECM expression).

### Mechanosensitivity and NF-κB activation

NF-κB nuclear localization activates transcription of related proinflammatory genes. Some evidence also suggest that NF-κB is mechanosensitive, where it is implicated in responses to mechanical stimulation in some cell types (*26*). NF-κB is responsive to substrate stiffness, where activation was temporarily induced in H1299 lung adenocarcinoma cells grown on a stiff substrate but not in cells grown on a soft substrate. Moreover, the ROCK inhibitor Y27632 reduced the activity of NF-κB in 2D cultured cells grown on stiff substrates (*28*). Our results show that, in 3D hydrogel environments, whose stiffness is on the same order of magnitude as the cell itself (i.e., soft, ~5-kPa stiffness), activation of RhoA with CN03 decreased NF-κB translocation under TNFα stimulation. These results indicate that increased contractility inhibits NF-κB translocation into the nucleus in rounded cells grown on physiologically relevant “soft” 3D environment. This response was quantified in thousands of cells using a novel high-throughput imaging flow cytometry and cell staining in situ, giving more rigor than traditional assays for NF-κB nuclear translocation (fluorescence microscopy) and more insight into the response of cells to biomimetic 3D cultures. We also show that activation of RhoA with CN03 inhibited TNFα-induced NF-κB pathway signaling. This relationship of decreased NF-κB activation/signaling with increased contractility differs from that observed for mechanosensitive transcription factors associated protein (YAP) and transcriptional coactivator with PDZ-binding motif (TAZ) and retinoic acid receptor γ (RARγ), where nuclear localization is promoted with increased stress, cytoskeletal contractility, and nuclear tension. YAP/TAZ tends to localize to the nucleus in high-tension cells cultured on stiff substrates (*29*). Mechanical stress also leads to nuclear localization of RARγ (*30*). Tension balance between the cytoskeleton and nucleus permits nuclear localization of transcription factor and its activation. However, in gelatinous ECMs, such as in the NP, and for cells grown within or on the surface of soft substrates, the lack of stress fibers alters the force balance and the relevant factors that regulate nuclear localization.

### TNFα-induced loss of cortical contractility: Consequences on cell mechanobiology

Studies have shown that human NP cells obtained from disc samples with histopathological degradation exhibit morphological changes such as development of cell extensions (*19*), although the driving forces for these morphological changes are unknown. Our current findings indicate that the cell morphological changes occurring with degradation could result from an inflammatory insult during disease. Our findings demonstrate a direct consequence of altered morphology on cell osmotic biophysical properties (*L*_p_). Hydraulic permeability is an important variable for NP function because cells are exposed to dynamic osmotic stimuli. Healthy IVDs have a higher osmotic environment of ~434 mOsm/liter (*31*), and cells are exposed to diurnal osmotic stress (*32*). In degeneration, the osmotic environment is altered by PG loss and lower water content (*2*). Here, we report the direct role of cortical contractility in the regulation of single-cell osmotic properties, showing that changes in hydraulic permeability of single NP cells are correlated with and regulated by specific components of the actomyosin regulatory network. To analyze NP cells’ biophysical properties in an environment that more closely preserves their native cell morphology, we performed experiments in 3D models (cells encapsulated within alginate beads and native ECM explants). In endothelial cells grown in 2D on glass substrate, TNFα induces stress fiber formation and cell elongation, as well as increased traction forces (*33*). However, NP cells in situ do not have stress fibers in their rounded morphology; therefore, to understand the specific mechanobiological events occurring with inflammation, there is a need to model that native rounded cellular morphology. In 3D cultured cells, we show that TNFα decreased cortical actomyosin contractility mediated by decreased RhoA, Rho GTPase signaling, myosin-II expression, and pMLC, which then morphologically manifests through the formation of cell extensions and cell polarization. This dysregulation of cortical actomyosin induced by TNFα was responsible for the alterations in biophysical properties, including increased hydraulic permeability and decreased elastic modulus. When RhoA was constitutively activated in the presence of TNFα, hydraulic permeability and cell modulus recovered to untreated levels.

In the loss-of-function experiments, the effects of actomyosin contractility inhibitors blebbistatin, Y27632, and ML7, which act on differing targets that participate in regulation of actomyosin contractility, were not uniform. The myosin-II inhibitor blebbistatin most closely phenocopied the biomechanical (hyperosmotic permeability and stiffness) and morphological effects observed with TNFα. The effects of the ROCK inhibitor Y27632 were milder than that of blebbistatin. Y27632 produced similar effects as TNFα on cell radius, pMLC, circularity, and elastic modulus but failed to significantly increase hydraulic permeability. Transcriptomic results revealed that TNFα significantly decreased the expression of *ROCK*, the best studied effector of RhoA, and a more novel effector CIT. However, cotreatment with CN03 + TNFα increased CIT and formin pathways but not ROCK pathway. This further supports the finding that ROCK is only partially contributing to the observed effects of TNFα and that other Rho GTPase effector mechanisms are at play in mediating downstream actomyosin contractility. We also found that MYLK does not significantly regulate NP cell hydraulic permeability, cell stiffness, or size. While the effect size of the inhibitors was not uniform, the significantly correlated change in permeability supports the premise that decreases in myosin-II–driven contractility are sufficient to increase hyperosmotic hydraulic permeability, while increases in contractility driven by constitutive activation of RhoA resulted in decreased permeability. Future studies into the mechanistic contributions of CIT- and formin-mediated actomyosin contractility may shed a more detailed mechanism by which RhoA is mediating pMLC in NP cell inflammatory environment. We also observed changes in *RhoD* expression that coordinated with the expression changes of *RhoA* in both TNFα and CN03 + TNFα, which may contribute to the observed changes in actomyosin contractility and permeability. The expression of *RhoJ*, a member of the Rho family that reduces actomyosin contractility (*34*), was significantly increased by TNFα, indicating that additional feedback mechanisms may be at play that can specifically reduce cortical actomyosin. We observed a greater effect of TNFα and blebbistatin on hyperosmotic permeability versus hypo-osmotic permeability, suggesting that disruption to actomyosin contractility has a bigger effect on cell shrinking rather than cell swelling. This may be because cell shrinking is an active process that requires intact cell contractility function, whereas cell swelling is more passive and not as dependent on actomyosin contractility. Since TNFα causes cells to swell basally, their ability to undergo further volume increase under hypo-osmotic stress is therefore limited. We observed that TNFα-treated cells are more likely to burst under hypo-osmotic conditions than untreated cells.

### Consequences for tissue homeostasis and ECM integrity

Inflammation is a key mediator of catabolic signaling and disc degradation. The role of TNFα in the induction of ECM degradation and its elevated expression in degenerated discs (*3*, *8*) places this cytokine as a central target to decipher the early molecular mechanisms of ECM degeneration. We observed that disruption to cellular morphology and biomechanics occurred early in the inflammatory cascade preceding ECM degradation, thus suggesting that alterations in cell morphology and biomechanics are early indicators of ECM degradation. PG synthesis has been reported to vary with cell size driven by osmotic stimuli, with higher synthesis observed under hyperosmotic conditions (430 mOsm/liter) that prevented cell swelling, compared to cells under free-swelling conditions at 280 mOsm/liter (*35*). The finding that RhoA activation blocked cell size increase (i.e., blocked cell swelling) may have caused a shift in cell metabolic responses favoring enhanced PG synthesis and contributing to ECM protection under inflammatory conditions. We find that NP cells from the CN03 + TNFα–treated group had higher ACAN and other ECM gene expression than TNFα-treated cells. Alterations to NF-κB activity may also confer additional protection to ECM integrity. Downstream targets of NF-κB signaling in IVD cells include many matrix-degrading enzymes [e.g., *ADAMTS4*, *ADAMTS5*, *MMP1*, *MMP2*, *MMP3*, *MMP9*, and *MMP13* (*25*)]. Blocking NF-κB activity has been shown to decrease matrix-degrading enzyme genes (e.g., *MMP3*) (*36*). However, in rabbit annulus fibrosis cells, NF-κB inhibition did not rescue the loss of PG or collagen synthesis in response to mechanical loading alone or in combined mechanical and inflammatory stimulation. Other attempts to block NF-κB have similarly failed to inhibit its activity using well-established pharmaceuticals (e.g., dexamethasone) or natural substances, challenging the efficacy of this therapeutic approach for the IVD (*25*). Therefore, our findings that RhoA activation blocks canonical NF-κB translocation and transcription, reduces noncanonical NF-κB signaling, and confers protection of ECM integrity represent a novel therapeutic strategy against TNFα-induced degradation. This finding is particularly interesting, namely, that basal actomyosin contractility is critical to ECM regulation, even in the absence of applied mechanical loading. It will be interesting to explore in future studies whether increasing contractility with loading will also confer mechanoprotection on cell and tissue integrity. Continued development of therapeutics that modulate cell contractility will aid in our understanding of cross-talk between mechanobiological and inflammatory signaling and in developing effective treatments for DD.

## MATERIALS AND METHODS

### Study design

This was a controlled laboratory experiment using tissue explants and cell cultures isolated from bovine NP, which is similar in size, cell type, and physicochemical environment to adult human lumbar NP. NP tissue and subsequent cell isolation were performed routinely (at least four times per year over the course of 2 years). Cells and tissues used in this study were sourced from approximately eight animals (bovine). Each cell isolation yields millions of cells, which were subdivided among experiments on an ongoing basis. Cells or tissue explants were randomly assigned into one of the following treatment groups: untreated, TNFα, CN03, CN03 + TNFα, blebbistatin, Y27632, or ML7. The sample size for these experiments varied on the basis of the outcome measure, as follows.

#### Amnis ImageStream

Each sample consisted of measurements from ≈15,000 cells per group for circularity and pMLC measurements and ≈5000 cells per group for NF-κB nuclear localization measurements was used. Replication: For each experimental group, circularity and pMLC intensity data were collected from three replicates assayed over the course of 3 days. An untreated control group was assayed during every experiment to control for interassay variability. For each experimental group, NF-κB nuclear localization data were collected from three replicates assayed on the same day.

#### Biophysical properties

A sample size of ≈150 to 200 cells per group was used for radius data, that of ≈10 to 15 cells per group was used for osmotic analysis measurements (hydraulic permeability and osmotically active water content), and that of ≈50 cells per group for elastic modulus measurements was obtained. Sample sizes for radius and osmotic analysis were based on the number of cells within the number of replicates performed. For elastic modulus data, the sample size was determined on the basis of the number for single-cell tests performed before significant cell spreading was noted in samples. Replication: For each experimental group, radius data were collected from 10 images (technical replicates) collected over the course of 2 days. For each experimental group, hydraulic permeability and osmotically active water content were collected from four replicates measured over the course of 4 days. For each experimental group, elastic modulus data were collected over 2 days of data collection. Biophysical property measurements from ML7-treated cells were performed using a different microscopy system compared to that used in other inhibitors or TNFα groups. Therefore, to account for variability due to changes in imaging system, an untreated control group was also analyzed for quality control. Mean values of untreated cells were compared between experimental setups (confocal and inverted microscope setups), and the data collected using the inverted microscope were scaled to the mean of the measurements made using the confocal system, generating a normalization factor, which ranged from 0.85 to 1.15, depending on the measurement. This scaling factor was applied to ML7 measurements and their corresponding control groups before subsequent statistical analyses.

#### RNA sequencing

RNA was isolated from three experimental replicates per group with 200,000 cells per sample.

#### Explant studies

A sample size of six to seven explants per group was used for explant GAG measurement data. For explant cell circularity measurements, a sample size of three explants per group and nine images per explant was used. The sample size for explant studies was based on feasibility of tissue isolation. Replication: Explant GAG content was collected from two independent experiments, with tissues isolated from two different animals.

### Cell isolation and culture

Lumbar spines from freshly slaughtered juvenile cows were obtained from an abattoir (Green Village Packing Company, Green Village, NJ, USA; permission was obtained to use these animal parts for research). NP tissue was dissected from multiple lumbar spinal levels and then pooled, minced, and digested in complete media [high glucose Dulbecco’s modified Eagle’s medium (DMEM) + 10% fetal bovine serum (FBS) + 1% antibiotic-antimycotic solution] supplemented with collagenase type I (0.3 mg/ml; Sigma-Aldrich) and collagenase type II (0.3 mg/ml; Sigma-Aldrich) for 3 hours at 37°C with gentle agitation. Cell digest was passed through a 70-μm cell strainer, washed, counted, and grown in complete medium. For 3D culture, passage 1 (P1) cells were trypsinized and placed in 1.2% alginate beads to promote round morphology.

### Alginate bead preparation

Alginate medium viscosity (1.2%; Sigma-Aldrich) was dissolved in 150 mM NaCl with 20 mM Hepes (pH 7.4) and sterilized by passing it through a syringe filter (pore size: 0.22 μm). After trypsinization, cells were washed with phosphate-buffered saline (PBS) and resuspended in alginate as 1 × 10^6^ cells/ml (approximately 25 to 35 μl of beads; 2.5 × 10^4^ to 3 × 10^4^ cells per bead). Beads were polymerized by placing drops of the alginate-cell mixture into a bath of 102 mM CaCl_2_ with 10 mM Hepes (pH 7.4) using a 21-gauge needle. Beads were allowed to polymerize in CaCl_2_ for 10 min at 37°C and then rinsed three times for 2 min each with 150 mM NaCl with 10 mM Hepes (pH 7.4). Cells in beads were cultured in complete medium overnight at 37°C before proceeding with treatments.

### Treatments

Recombinant rat TNFα (R&D Systems, Minneapolis, MN, USA) was prepared in sterile distilled H_2_O with 0.1% bovine serum albumin (BSA) and diluted in DMEM before use. Beads were exposed to the following treatments for 24 hours in complete medium: (i) untreated, (ii) TNFα (10 ng/ml), (iii) 10 μM blebbistatin (myosin-II inhibitor), (iv) 10 μM Y27632 (ROCK inhibitor), (v) 10 μM ML7 (MYLK inhibitor), and (vi) CN03 (1 μg/ml; Rho activator) ± TNFα (10 ng/ml). For experiments with blebbistatin, Y27632, or ML7 treatments, control groups were also treated with 0.1% dimethyl sulfoxide to control for the solvent used. In group (vi) samples, CN03 was administered 60 min before TNFα stimulation to allow intracellular activity before stimulation.

### Immunofluorescence

For immunofluorescence analysis of cells cultured in beads, all solutions were prepared in Hanks’ balanced salt solution (HBSS + 1.26 mM CaCl_2_ + 400 μM MgSO_4_). Beads were fixed in 4% paraformaldehyde for 10 min at room temperature. Cells were permeabilized in HBSS + 0.5% Triton X-100 for 5 min, blocked in HBSS + 1% BSA for 1 hour. Cells were stained with Alexa Fluor 555–phalloidin (1:1000; Abcam) or primary antibodies, anti–β-tubulin (1:1000; Sigma-Aldrich) or anti-vimentin (1:1000; Abcam), overnight at 4°C, followed by 1-hour incubation with anti-mouse Alexa Fluor 488 (1:500; Molecular Probes). After three washes with HBSS + 0.1% Tween 20, cells were released from beads with 55 mM sodium citrate with 10 mM Hepes (pH 7.4) for 10 min at room temperature, placed in poly-l-lysine–coated glass coverslips, and mounted with ProLong (Molecular Probes). Images were taken with a laser scanning confocal microscope Olympus XL70 using a 40× objective as a Z-stack with 1-μm space between slices. For cells in monolayers, all solutions were prepared in PBS. Cells were grown in poly-l-lysine–coated coverslips for 24 hours and fixed in 4% paraformaldehyde for 10 min at room temperature. Cells were permeabilized in 0.1% Triton and blocked with 5% BSA. Cells were stained with Alexa Fluor 555–phalloidin (1:1000; Abcam) or primary antibodies anti–β-tubulin (1:1000; Sigma-Aldrich) or anti-vimentin (1:1000; Abcam) overnight at 4°C, followed by 1-hour incubation with anti-mouse Alexa Fluor 488 (1:500; Molecular Probes). Coverslips were mounted with ProLong. Images were taken with the laser scanning confocal microscope Olympus XL70. Confocal images were processed in ImageJ (National Institutes of Health). A Z-projection with maximum intensity was generated. Thresholding was used to subtract background during image analysis.

### Imaging flow cytometry

Alginate beads were fixed, permeabilized, and blocked as described above. Beads were stained with anti-pMLC (1:100; Cell Signaling Technology) overnight at 4°C, followed by anti-rabbit Alexa Fluor 488 (1:500; Molecular Probes) for 1 hour at room temperature, or with anti–NF-κB–p65 (1:50; Thermo Fisher Scientific) overnight at 4°C, followed by anti-mouse Alexa Fluor 568 (1:1000; Molecular Probes) for 1 hour at room temperature. After staining, cells were released from beads, counterstained with Draq5 nuclear stain, and analyzed using an Amnis ImageStream X Mark II imaging flow cytometer, which images individual cells in a high-throughput manner. Images were analyzed with Amnis IDEAS software. Brightfield images are first segmented automatically in IDEAS software to determine cell masks for each individual cell. These masked regions are subsequently used for the determination of cell size and shape features and also provide the area within which fluorescent markers are observed. Cells are first gated for single cells using mask size and aspect ratio to eliminate small cell fragments, debris, and large multicellular clusters. Cell images are then gated to remove out-of-focus images. Last, cells are gated for under- and over-range fluorescence intensity for all markers used. For pMLC quantification, pMLC intensities of individual cells were normalized to cell size as determined by corresponding brightfield images and then reported normalized to untreated control per experiment to account for interexperimental biological variability. For NF-κB nuclear localization, a similarity score between NF-κB and the nuclear stain per cell was extracted from IDEAS software and was reported normalized to the similarity score for the untreated group.

### Osmotic properties and cell size

All osmotic swelling and shrinking solutions were prepared with NaCl. Cells were released from alginate beads and resuspended in DMEM. Forty microliters of cell suspension was seeded into a custom-made Y-shaped osmotic loading microfluidic chamber. The polydimethylsiloxane channel (1-cm length, 300-μm width, 200-μm height) was sealed over a poly-d-lysine–treated glass slide. Cells were incubated at 37°C for 30 min to allow attachment while retaining a round morphology. Cells were equilibrated at 333 mOsm/liter for 5 min through the addition of 100 μl of 333-mOsm solution to each of the two upstream wells of the osmotic chamber. Loading was performed at room temperature to observe passive osmotic properties of the cells, as it has been previously shown that cells do not exhibit active volume recovery when loaded in NaCl under these conditions and experimental duration. After equilibration, a single hyperosmotic loading step was applied (333 to 466 mOsm) followed by a hypo-osmotic loading step (466 to 333 mOsm) with 5-min equilibration time between steps. These concentrations were selected on the basis of typical osmolarities found within the disc in vivo. During osmotic loading, cells were imaged on an inverted confocal microscope with DIC filter. Images (0.2 μm per pixel) were acquired at a frequency of 0.5 Hz. ML7-treated cells were imaged on an inverted brightfield microscope with images (0.4 μm per pixel) acquired at a frequency of 0.5 Hz. A MATLAB routine was used to segment individual cells and to calculate cell radius and the volume response over time per cell (*17*). Volume response per cell was curve-fitted using multiphasic mixture theory to compute the following material properties: hydraulic permeability (*L*_p_) and osmotically active intracellular water content (φ*_ir_*) (*17*, *22*, *23*, *37*).

### Analysis of volume response

The volume response of cells to osmotic loading was analyzed as previously described (*17*) with the Kedem-Katchalsky model, formulated using equations based on a mixture theory framework (*n* = 10 to 15 cells per osmotic step) (*22*, *23*, *37*). NaCl was modeled as a nonpermeating solute at room temperature (*37*), simplifying the differential equation governing cell volume response *V*(*t*) to

$$\frac{\mathrm{dV}}{\mathrm{dt}}=AL_{p}R\theta (c_{i}-c_{e})$$ (1)

where *A* is the volume-dependent cell surface area ($A=3\frac{V}{a}$, *a* is the cell radius), *L*_p_ is hydraulic permeability, *R* is the universal gas constant, θ is the absolute temperature, and *c*_i_ and *c*_e_ are the intracellular and extracellular osmolarities, respectively. Experimentally, the extracellular osmolarity was defined by step application of NaCl solutions. The intracellular osmolarity is related to the number of moles of solute within the cell (*n*_i_) and the cell volume by

$$c_{i}=\frac{n_{i}}{\varphi_{i}V},\frac{{\mathrm{dn}}_{i}}{\mathrm{dt}}=0$$ (2)

where φ_i_ is the volume fraction of osmotically active water in the cytoplasm. As the cell expands, this volume fraction changes as water enters the cell according to the principle of conservation of mass

$$\phi_{i}=1-(1-\phi_{\text{ir}})\frac{V_{r}}{V}$$ (3)

where the subscript r denotes the reference state (before each loading step). The reference intracellular water content φ*_ir_* can be deduced from these equations in the limit when $\frac{\mathrm{dV}}{\mathrm{dt}}\to 0$ (i.e., after the cell reaches equilibrium volume) from the equation

$$\frac{V_{\infty }}{V_{r}}=1-\phi_{\text{ir}}+\frac{\phi_{\text{ir}}c_{\text{er}}}{c_{e}}$$ (4)

The measured cell volume response was curve-fitted to these equations using a custom MATLAB routine to calculate the parameters φ*_ir_* and *L*_p_ for each cell at each osmotic loading step.

### Atomic force microscopy

Cells were released from beads as previously described just before testing, placed in a poly-d-lysine–treated petri dish, and heated biochamber for 30 min to allow cell adhesion while retaining a round morphology (DMEM). Cells were tested using an Asylum MFP-3D AFM with a spherical polystyrene tip probe (3-μm radius). AFM probe tip was visually aligned, and its location was saved within an AFM software to allow for precise positioning of tip over cells during testing. Single force-indentation curves obtained per cell were fitted to a Hertz model of elasticity to determine the elastic modulus using a built-in Asylum AFM software.

### RNA sequencing

For RNA-seq experiments, bovine NP cells were seeded in a 12-well plate (200,000 cells per well) and treated overnight before RNA isolation. Total RNA from each group (*n* = 3 per group) was extracted using an RNEasy kit (QIAGEN, Valencia, CA). RNA sample quality was confirmed using Bioanalyzer (Agilent); all samples had RNA integrity numbers of ≥9.7. Poly-A tail pulldown was then performed to enrich mRNAs from total RNA samples. Libraries were constructed using the Illumina TruSeq Stranded mRNA Kit and sequenced at the Columbia Genome Center on Illumina NovaSeq 6000 with paired-end 100–base pair reads for each sample. Real Time Analysis (RTA) (Illumina) was used for base calling and bcl2fastq2 (version 2.19) for converting BCL to fastq format, coupled with adaptor trimming. A pseudo-alignment to a kallisto index created from a reference transcriptome (*Bos taurus*: ARS-UCD1.2) was then performed using kallisto (0.44.0). DESeq2, an R package for differential expression analysis, was used to test for differentially expressed genes between two experimental groups using RNA-seq counts data. Significant differentially expressed genes were determined as those with an adjusted *P* value (*P*_adj_) of <0.05. Select differentially expressed genes were presented as heatmaps organized on the basis of the KEGG pathways for TNFα inflammatory response and NF-κB activation, as well as for genes related to ECM or matrix-degrading enzymes. Volcano plots and dendrograms of significant differentially expressed genes were also generated. Transcriptome pathway analysis was performed using significant differentially expressed genes and Reactome pathway analysis software (*38*).

### Western blot

For NF-κB Western blot analysis, cells in monolayers were cultured in DMEM + 1% FBS + 1% antibiotic-antimycotic solution for 24 hours before treatment. Treatments with TNFα and CN03 were performed as described above but in the absence of serum. After 10, 30, and 60 min, nuclear and cytoplasmic protein fractions were collected using the NE-PER Nuclear and Cytoplasmic Extraction Kit (Thermo Fisher Scientific). Nuclear fraction lysates were loaded in 8% tris-glycine SDS–polyacrylamide gel electrophoresis and transferred to nitrocellulose. Blots were blocked and stained with anti–NF-κB–p65 (1:1000; Cell Signaling Technology) and anti–lamin a/c (1:1000; Cell Signaling Technology) overnight at 4°C, followed by anti-mouse or anti-rabbit horseradish peroxidase–conjugated secondary antibodies (1:1000; Cell Signaling Technology) for 1 hour at room temperature. Blot was imaged using an Azure C600 imaging system (Azure Biosystems).

### NP explant isolation and organ culture

Tails from freshly slaughtered adult cows were used for NP explant isolation. Full-thickness cylindrical NP explants were obtained with 4-mm surgical punches. For the swelling-restricted culture system, a 2% (w/v) low-melt type VII agarose (Sigma-Aldrich) was dissolved in PBS and sterilized using an autoclave. The NP explants were centered in the middle of each well in a 24-well tissue culture plate, and hydrogel solution was cast over the explants until covered and then allowed to polymerize. Complete medium (high glucose DMEM + 10% FBS + 1% antibiotic-antimycotic solution) was then added onto the explant in hydrogel and was cultured under standard culture conditions (5% CO_2_, 37°C). For validation of the culture system, explant-in-hydrogel samples (i.e., swelling-restricted explants) were compared to those cultured under free-swelling conditions (i.e., in complete media with no restriction on swelling) for GAG loss and NO release into the media (see Supplementary Materials and Methods). For treatment effects, explants in swelling-restricted culture system were treated with TNFα (10 ng/ml) ± CN03 (1 μg/ml) in complete media for 14 days.

### Explant GAG content

Explants were collected, weighed to determine wet weight, lyophilized overnight in a vacuum desiccator, and reweighted to determine both tissue dry weight and water content. Samples were digested overnight in papain (0.3 mg/ml) in 100 mM sodium acetate, 10 mM cysteine HCl, and 50 mM EDTA. DMMB assay was used to quantify GAG content of tissue digests (*39*). GAG content per tissue wet weight was normalized to untreated control.

### Histology

Explants were collected, fixed in 4% paraformaldehyde for 24 hours, and processed for paraffin embedding using standard methods. Five-micrometer sections were stained with Alcian blue (pH 1.0) to determine GAG distribution. Images were obtained using a transmitted light microscope (Olympus BX53 Microscope, QImaging camera and 20× objective).

### Circularity analysis of NP cells in explants

NP explants were cultured in complete media with or without TNFα and with or without CN03 for 2 weeks. Explants were removed from agarose casts and fixed in 4% paraformaldehyde for 24 hours. Tissue was cut in thin sections with a scalpel, and sections were then permeabilized with 0.1% Triton for 15 min and stained with Alexa Fluor 555–phalloidin (1:100; Abcam) overnight at 4°C. Tissue sections were imaged with a Nikon Ti Eclipse inverted confocal microscope using a 10× objective. Images were processed in ImageJ to determine the circularity of cells within an explant.

### Statistical analysis

For data collected via the ImageStream imaging flow cytometry (pMLC intensity, circularity, and NF-κB nuclear localization), data distributions were compared using two-sample Kolmogorov tests that were performed with *P* < 0.05 considered as significant. Distributions of cell circularity measured in explants were also compared using two-sample Kolmogorov tests with *P* < 0.05 considered as significant. Comparison of biophysical properties (cell radius, hydraulic permeability, osmotically active water content, and elastic modulus) between untreated and TNFα-treated cell populations was performed using two-tailed *t* tests with *P* < 0.05 set as significant (Statistica). Comparison of biophysical properties between untreated versus inhibitor groups (blebbistatin, Y27632, and ML7) and TNFα versus inhibitor groups were performed using two-tailed *t* tests with *P* < 0.05 set as significant (Statistica). For comparison of biophysical properties, explant GAG content, and NF-κB nuclear localization for effects of TNFα ± CN03 (untreated, TNFα, CN03, and CN03 + TNFα), analyses of variance (ANOVAs) were performed with *P* < 0.05 set as significant. Direct comparisons between groups were performed using Fisher’s least significant difference post hoc test with *P* < 0.05 set as significant (Statistica). For RNA-seq data, Benjamini-Hochberg adjusted *P* values were obtained from DESeq2 analysis. Significant differentially expressed genes presented in heatmaps and used for pathway analysis were determined as those with *P*_adj_ < 0.05. To examine correlations between biophysical properties, linear regression was performed to determine *R*^2^ and *P* values, with *P* < 0.05 set as significant (Statistica). The dataset used in the correlation analysis was based on the average value of actomyosin contractility (pMLC staining intensity), hydraulic permeability, and circularity per treatment group.

## Supplementary Material

aba2368_SM.pdf

## SUPPLEMENTARY MATERIALS

Supplementary material for this article is available at http://advances.sciencemag.org/cgi/content/full/6/34/eaba2368/DC1

## Appendix

View/request a protocol for this paper from *Bio-protocol*.
