# Supplementary material for: Actomyosin contractility confers mechanoprotection against TNFα-induced disruption of the intervertebral disc
Source: Sci Adv. 2020 Aug 19;6(34):eaba2368. doi: 10.1126/sciadv.aba2368 (PMC7438088; doi:10.1126/sciadv.aba2368)
Supplement: aba2368_SM.pdf [file aba2368_SM.pdf]

Science Advances
AAAS

advances.sciencemag.org/cgi/content/full/6/34/eaba2368/DC1

Supplementary Materials for

Actomyosin contractility confers mechanoprotection against TNFα-induced disruption of the intervertebral disc

Paula A. Hernandez, Timothy D. Jacobsen, Nadeen O. Chahine*

*Corresponding author. Email: noc7@columbia.edu

Published 19 August 2020, *Sci. Adv.* **6**, eaba2368 (2020)
DOI: 10.1126/sciadv.aba2368

This PDF file includes:

Supplementary Materials and Methods
Figs. S1 to S8

Supplementary Materials:

Materials and Methods

Immunofluorescence: For immunofluorescence analysis of cells cultured in beads, all solutions were prepared in Hanks' Balanced Salt Solution (HBSS + 1.26 mM $CaCl_2$ + 400 µM $MgSO_4$). Beads were fixed in 4% paraformaldehyde for 10 min at room temperature. Cells were permeabilized in HBSS + 0.5% Triton for 5 min, blocked in HBSS + 1% BSA for 1 hour. Cells were stained with Alexa 555-phalloidin (1:1,000 Abcam) or anti-vimentin (1:1,000, Abcam) overnight at 4°C, followed by 1 hour incubation with anti-Mouse Alexa 488 (1:500, Molecular Probes). After 3 washes with HBSS + 0.1% Tween-20, cells were released from beads with 55mM sodium citrate with 10 mM HEPES, pH 7.4 for 10min at room temperature, placed in poly-L-lysine coated glass coverslips and mounted with Prolong (Molecular Probes). Images were taken with laser scanning confocal microscope Olympus XL70 using a 40x objective as a Z-stack with 1 µm space between slices. For circularity measurements actin image threshold was adjusted for each image to subtract background during image analysis. Measurements of cell circularity in ML-141 treatment groups were done using "Analyze particles" in ImageJ and were normalized to untreated control.

Live/Dead Staining: Explants were removed from culture after 7 or 14 days and stained with 10µM Calcein AM (live cell stain) and 2µM Ethidium Homodimer-1 (dead cell stain) in PBS for 30 minutes at 37°C. Explants were imaged on a confocal microscope Olympus XL70 using 10x objective.

GAG and NO release: To validate the swelling constrained culture system, GAG and NO release into the media was measured and compared to free-swelling conditions. After 14 days in culture, media was collected and stored at -20°C. GAG content of the supernatant was measured using Blyscan Glycosaminoglycan Assay (Biocolor), while NO was measured using Griess Reagent System (Promega). Both values were normalized to explant size.

ML-141 treatment: ML141 was prepared in DMSO following manufacturer's instructions at a stock concentration of 10 mM. Beads were exposed to the following treatments for 24 hours in complete medium: (i) untreated, (ii) 10 ng/ml TNFα, (iii) 10 µM ML-141 (Cdc42 inhibitor), and (iv) 10 µM ML-141 + 10 ng/ml TNFα. Untreated included same 0.1% DMSO as ML-141 treatments. In co-treatment, ML-141 was added 30 min prior TNFα.

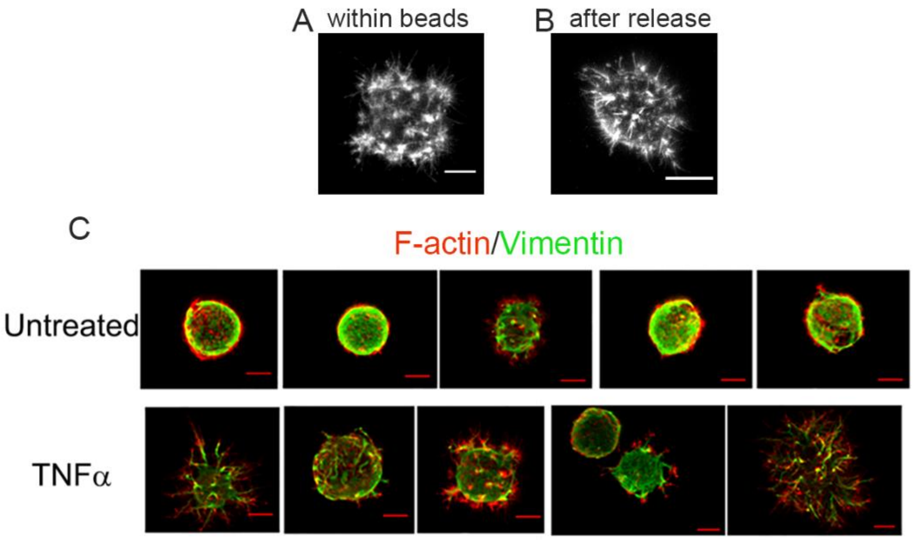

Fig. S1: Validation of cell removal from beads and the effect of reduced serum. Releasing cells from beads prior to confocal imaging does not alter actin structure. NP cells in alginate beads were treated with TNFα for 24h, fixed and stained within beads with phalloidin to visualize F-actin. A) cells were imaged within beads. B) cells were imaged after releasing them from beads. Scale bars are 10 μm. C) Reduced serum enhances the effect of TNFα. Representative confocal images of cells in alginate beads grown in 1% FBS and stained for F-actin (red) and vimentin (green). Z-projections with max intensity showed enhanced development of cell processes when incubated with 10 ng/ml TNFα for 24 hours. Scale bars are 10 μm.

A

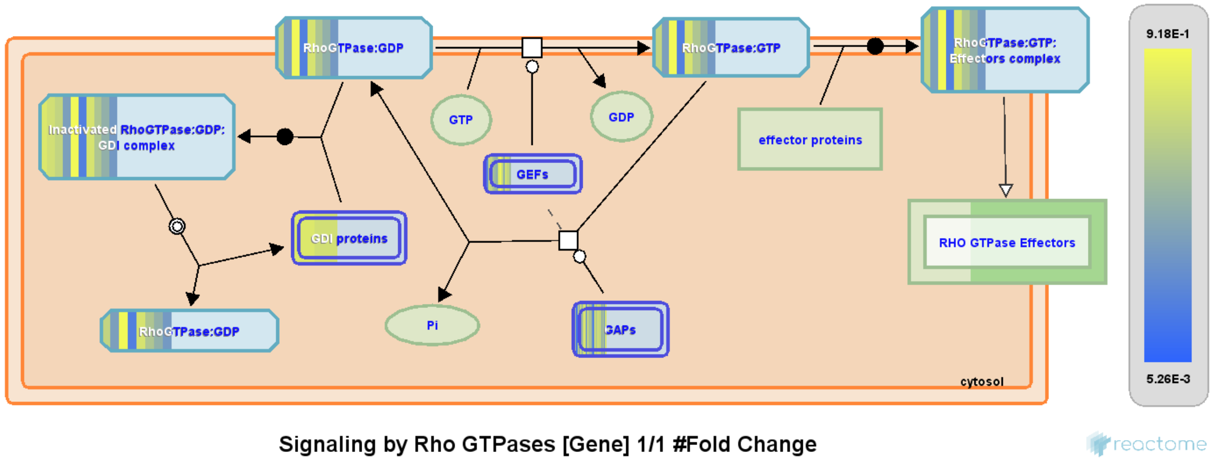
RhoGTPase:GDP
RhoGTPase:GTP
RhoGTPase:GTP:
Effectors complex
Inactivated RhoGTPase:GDP:
GDI complex
GTP
GDP
GEFs
effector proteins
GDI proteins
RHO GTPase Effectors
Pi
GAPs
RhoGTPase:GDP
cytosol
9.18E-1
5.26E-3
Signaling by Rho GTPases [Gene] 1/1 #Fold Change
reactome

B

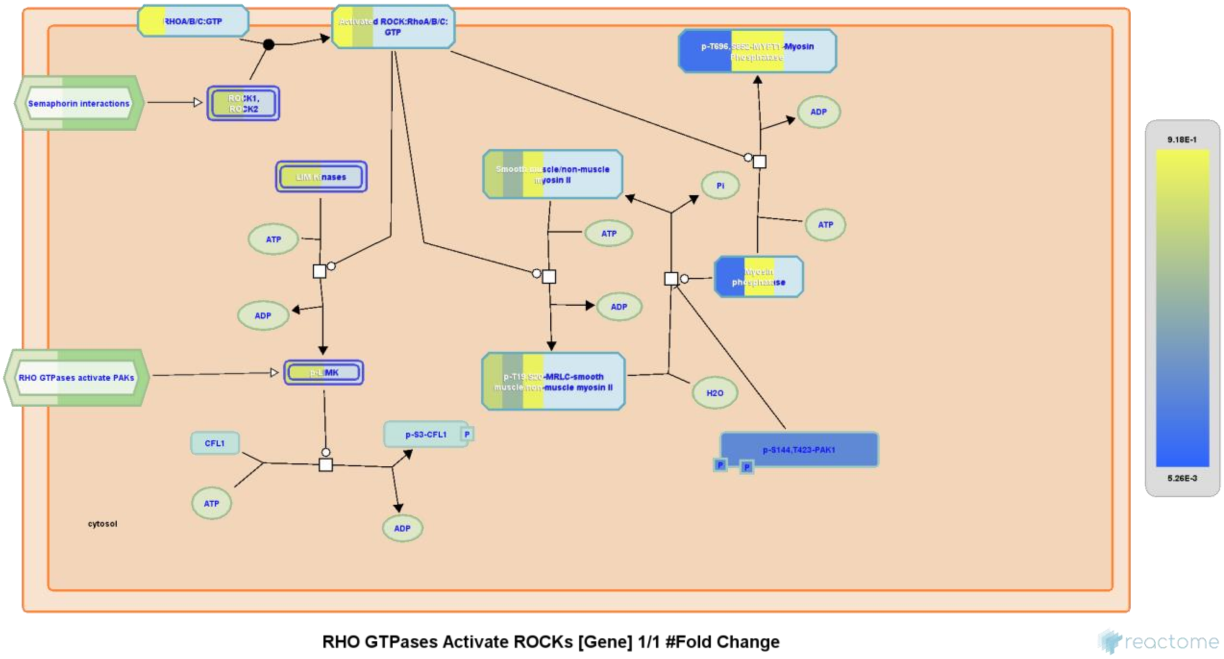
RHOA/B/C:GTP
Semaphorin interactions
ADP
ATP
Pi
H2O
CFL1
p-S3-CFL1
P
p-S144,T423-PAK1
RHO GTPases activate PAKs
cytosol
9.18E-1
5.26E-3
RHO GTPases Activate ROCKs [Gene] 1/1 #Fold Change
reactome

C

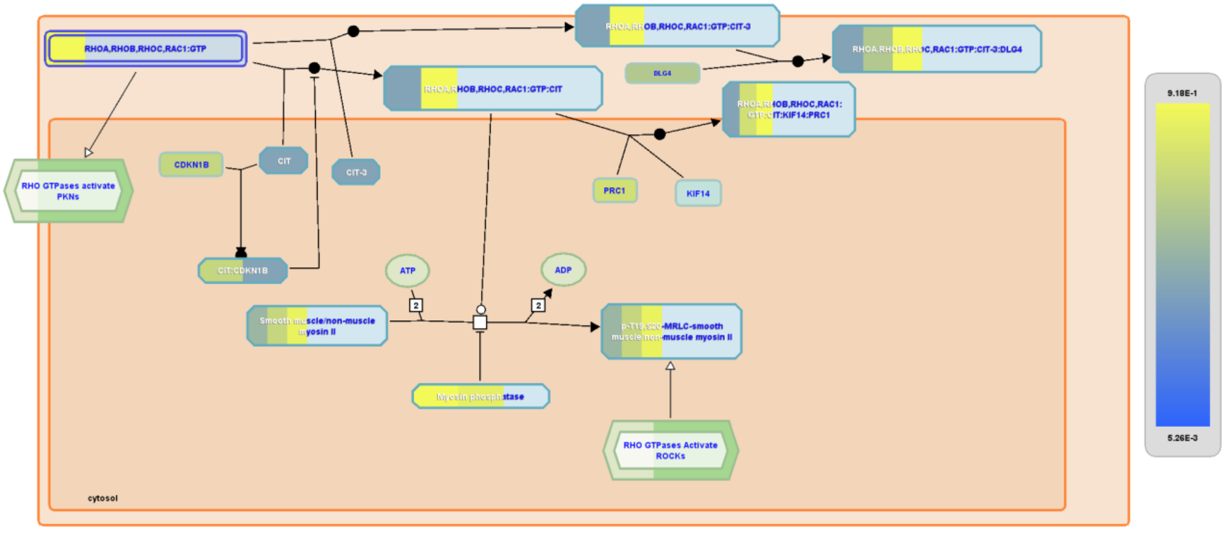

RHO GTPases activate CIT [Gene] 1/1 #Fold Change

D

Pathway name	#Entities found	#Entities total	Entities ratio	Entities pValue	Entities FDR
RHO GTPases activate CIT	11	23	0.00158467	0.07746182	0.79642074
Signaling by Rho GTPases	147	457	0.03148684	0.11482877	0.79642074
RHO GTPases Activate ROCKs	9	24	0.00165357	0.26751669	0.79642074
Molecules associated with elastic fibres	25	38	0.002618	2.06E-04	0.155422
Extracellular matrix organization	132	329	0.022668	2.07E-04	0.155422
Elastic fibre formation	28	46	0.003169	3.08E-04	0.155422
Collagen formation	48	104	0.007165	0.001653	0.297573
Collagen biosynthesis and modifying enzymes	37	76	0.005236	0.002243	0.372398

E List of significantly decreased entities (i.e. genes) found within pathway:

RHO GTPases activate CIT

PPP1CB;PPP1R12A;CDKN1B;P4HA1;FAP;DLG4;PRC1;MYH9;MYL6B;RHOA;CIT

Signaling by Rho GTPases

CYFIP2;NCKAP1;PHLDB1;FAM13B;WIPF1;MAML1;NRK;ITSN1;FAM13A;BUB1B;ARHGAP39;MYLK;GLS;SYDE2;EDC4;PPP1CB;CDC20;PPP2R1A;KIF5A;NIPAL3;ARHGDIB;EPC2;PRKACA;PDK1;GPT2;P3H4;PRKC

A;PIAS1;EEF1A1;SGO2;HIST1H2BB;HDGFL2;HIST1H2BD;PFN2;NOTCH1;HVCN1;STK39;ARHGAP18;SLC41A1;PIK3R2;IQGAP2;PRKCZ;IQGAP3;PMF1;RHOBTB1;ARHGAP22;INPP5B;ARHGAP21;KLC4;ARHGAP20;RACGAP1;RAD21;DVL2;RHPN1;SSR1;DVL3;SRGAP3;HOXC9;TRMT1L;SLC38A4;HOXC6;STARD13;ARHGEF37;ABCA5;FUCA1;ARHGEF39;H3F3A;PLK1;ARHGAP29;BRAF;ARHGAP26;NDC80;NET1;ARHGAP33;EIF2S3;DIAPH3;P4HA1;DLG4;HOOK1;DNAJC10;NOXA1;CNTN1;TUBA8;ITGB1;CDKN1B;MEGF10;RASGRF2;GDI2;MYL6B;PTPRK;CPZ;ARHGAP6;PKD1;HINT2;ATP7B;H2AFJ;NUF2;TIMP2;SPEG;RAC3;NDEL1;ARHGEF10;SEC13;PPP1R12A;PLEKHG5;H2AFV;PPP2R5A;PPP2R5D;PPP2R5C;RHOF;ELOVL7;ZFX;CKAP5;RHOD;NIPSNAP1;RHOA;CIT;GREM1;ARHGAP10;ARHGEF9;TUBB2B;TUBB2A;BIRC5;MYH9;KIF2C;EVL;RHOU;ALDH9A1;PRKAA2;ROCK1;NOXO1;TYMS;CENPA;EHBP1;FGD4;PAK1;PALMD;SV2A;MAPK3;ZSWIM3;LIMK2;BAIAP2;ACVR2A;CENPE;GALE;TPPP3;ABI2;FAP;PRC1;DLC1;ALMS1;CYP20A1;CTNNB1;PKN2;ESYT2;EIF3E;TRIM32;SPC24;ZNF696;R3HDM2;MAD2L1

RHO GTPases Activate ROCKs

PPP1R12A;ROCK1;MEGF10;LIMK2;MYL6B;TYMS;RHOA;PPP1CB;PAK1;P4HA1;FAP;MYH9;SLC38A4

Molecules associated with elastic fibres

ITGB1;FBN2;PRKAA2;ITGB5;ELN;LTBP4;LRP5;FBLN1;LTBP3;GXYLT2;PCDH18;FBLN2;FBLN5;CYTH2;EFEMP2;EFEMP1;TMEM204;ZNF207;TGFB2;ADPRHL1;JUP;TGFB3;FN1;CFAP410;GDF5;BMP4;MFAP4;COL4A1;MFAP2

Extracellular matrix organization

APP;COL16A1;SPARC;COL12A1;LOXL3;TNC;INPPL1;PLOD3;GXYLT2;PLOD1;ALDH1L2;LOXL1;COMP;LAMP5;EFEMP2;EFEMP1;CAPN6;CAPN7;BSG;ZNF207;ARID2;PRKACA;CAST;COL27A1;ADPRHL1;PHYKPL;P3H2;P3H1;PRKCA;P3H3;CFAP410;HSPG2;LOX;COL4A2;OPTC;COL4A1;ADAM12;COL8A2;COL4A5;COL8A1;ATF7;KCTD12;PPIB;FBN2;NDRG4;SDC3;LTBP4;HTRA1;PDGFA;FBLN1;LTBP3;EFNA4;PCDH18;NID2;FBLN2;RHOBTB1;FBLN5;ARHGAP20;COMMD9;TSPAN6;SERPINH1;MBIP;TRMT1L;TGFB2;CRTAP;JUP;TGFB3;FN1;GDF5;NDC80;PRRC2B;SLC16A13;COL1A1;BMP4;SLC6A8;COL1A2;P4HA1;COL5A1;P4HA2;P4HA3;ITGA10;COL5A2;ITGA11;ITGB1;PTPRS;ITGB5;COL14A1;SLC44A2;ELN;RASGRF2;ITGB2;SLC2A1;ADRA1D;CTSV;HAPLN1;ADAMTS2;TIMP2;CTSD;FGB;DST;MMP2;INSRR;BGN;CKAP4;ASPN;EEF1G;MMP11;MMP14;COL2A1;MMP16;MMP15;MATN1;PECAM1;COL6A3;ITGA7;MYH9;METTL26;DDR2;PLEC;LAMA5;COL15A1;PRKAA2;WDR26;LAMA2;COL11A1;COL11A2;LRP5;HSD17B4;NR2C2;THBS1;THBS3;ACAN;CYTH2;TTR;PALMD;TMEM204;NCAM1;JAM3;LAMB1;MFAP4;SH3D21;MFAP2;P4HB;FMOD;MEGF8

Elastic fibre formation

ITGB1;FBN2;PRKAA2;ITGB5;ELN;LTBP4;LOXL3;LRP5;FBLN1;LTBP3;GXYLT2;PCDH18;FBLN2;FBLN5;LOXL1;CYTH2;EFEMP2;EFEMP1;TMEM204;ZNF207;TGFB2;ADPRHL1;JUP;TGFB3;FN1;CFAP410;GDF5;BMP4;MFAP4;LOX;COL4A1;MFAP2

Collagen formation

COL16A1;COL14A1;RASGRF2;COL12A1;LOXL3;SLC2A1;TNC;INPPL1;PLOD3;ADRA1D;PLOD1;CTSV;LOXL1;LAMP5;ADAMTS2;ARID2;COL27A1;DST;P3H2;P3H1;P3H3;CKAP4;EEF1G;COL2A1;COL4A2;LOX;COL4A1;COL8A2;COL6A3;COL4A5;COL8A1;PPIB;METTL26;PLEC;COL15A1;COL11A1;COL11A2;COMMD9;PALMD;SERPINH1;CRTAP;COL1A1;COL1A2;SH3D21;P4HA1;COL5A1;P4HA2;P4HA3;COL5A2;P4HB;MEGF8

Collagen biosynthesis and modifying enzymes

COL15A1;COL16A1;COL14A1;RASGRF2;COL11A1;COL11A2;COL12A1;SLC2A1;PLOD3;ADRA1D;PLOD1;ADAMTS2;PALMD;SERPINH1;ARID2;CRTAP;COL27A1;P3H2;P3H1;P3H3;CKAP4;COL1A1;EEF1G;COL1

A2;COL2A1;SH3D21;COL5A1;COL4A2;P4HA1;COL4A1;P4HA2;P4HA3;COL5A2;COL8A2;COL6A3;COL4A5;COL8A1;P4HB;PPIB;METTL26

Fig. S2: Reactome pathways with significantly (padj<0.05) decreased target gene expression in TNFα vs. untreated groups. Reactome pathway for A) Signaling by Rho GTPases genes; (B) Rho GTPases ROCK pathway; (C) Rho GTPases CIT pathway. Color bar indicates fold change in TNFα vs. untreated. (D) Quantitative statistics from Reactome pathway analysis and (E) list of significant entities (i.e. genes) found within each pathway.

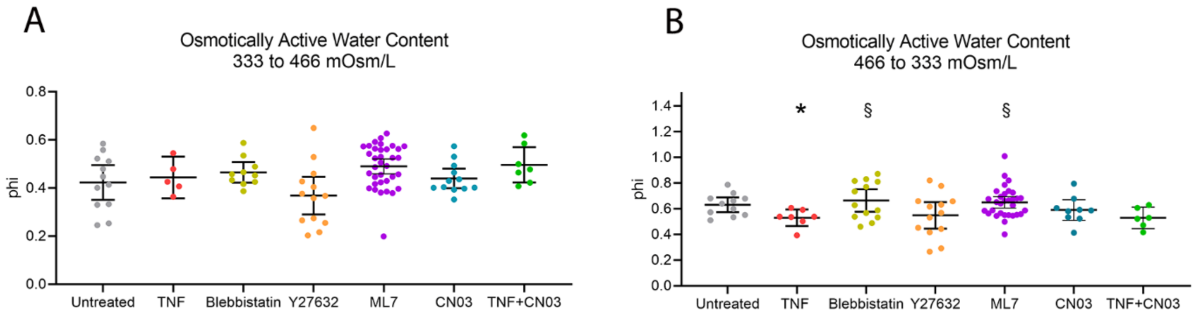

Fig. S3: Effect of drugs in osmotically active water content. (A) In the hyper-osmotic loading step, none of the drugs or TNFα were significantly different from untreated. (B) In the hypo-osmotic loading step, TNFα slightly but significantly decreased phi (φ_{ir}). Measurement from Blebbistatin and ML7 groups were significantly different from TNFα, but not different from control. §$p<0.05$ compared to TNFα. * $p<0.05$ compared to untreated.

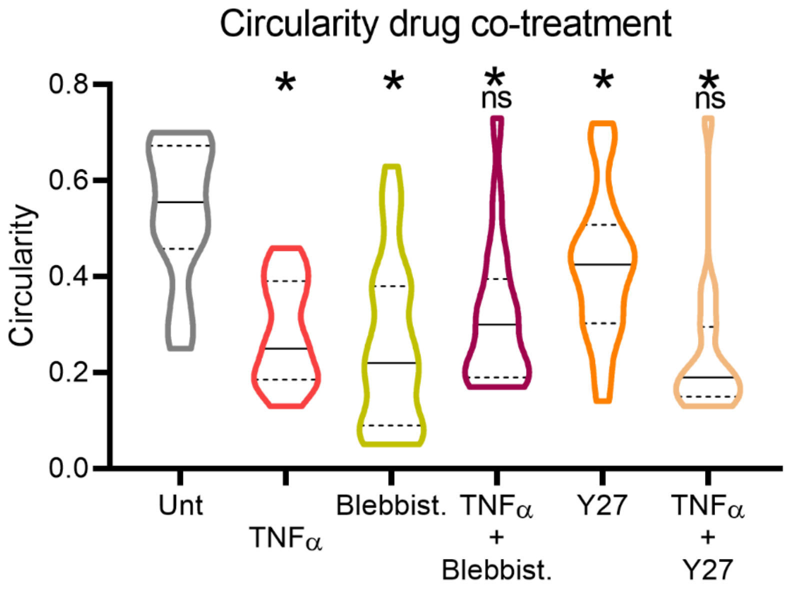

Fig. S4: Co-treatment of contractility-inhibitor drugs with TNFα does not further reduce circularity. Violin plot showing that co-treatment of Blebbistatin with TNFα or Y27632 with TNFα does not affect circularity more than the individual treatments (ns= not significant compared to TNFα). Measurements were done in ImageJ. Solid lines represent median and dotted lines represent quartiles. *p<0.05 compared to untreated.

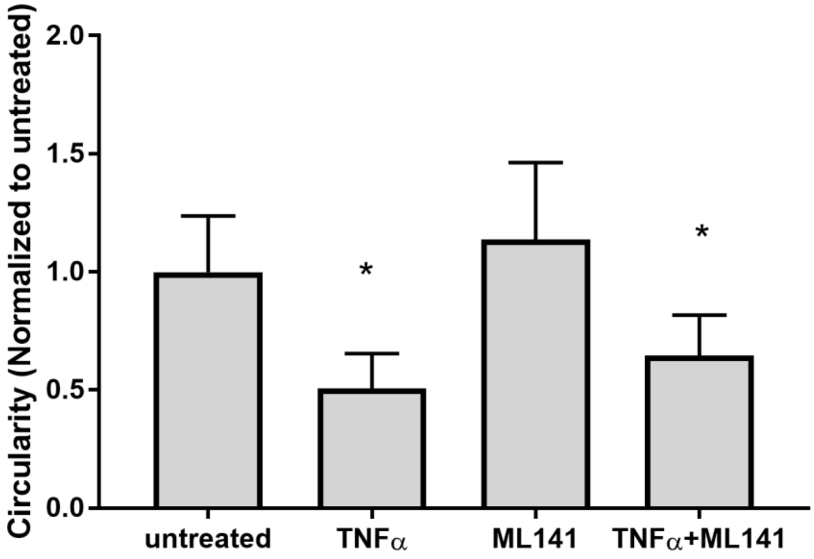

Fig. S5: Cdc42-GTPase inhibition does not protect against TNFα induced alteration to cell circularity. Co-treatment with the Cdc42-GTPase inhibitor ML141 (10 μM) did not protect against alterations in circularity or prevent formation of cell extensions (p=0.53). ML141 alone resulted in comparable circularity to untreated (p=0.29). *p<0.0005 vs. untreated. No significant difference between TNFα and ML141 + TNFα groups. Circularity of NP cells was determined using actin staining (phalloidin) and imaging. Circularity measurements were quantified using ImageJ. (N_{Unt}=16; $N_{TNF\alpha}$=9; N_{ML141}=45; $N_{ML141+TNF\alpha}$=24 cells).

A

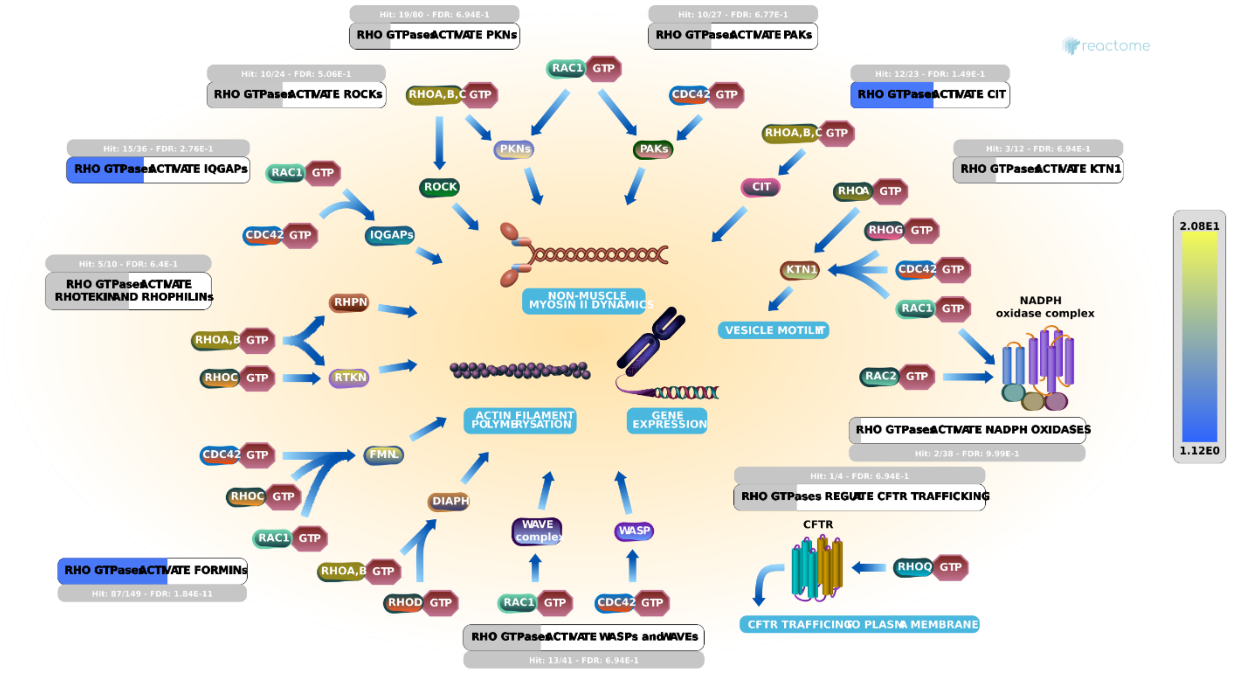
Hit: 19/80 - FDR: 6.94E-1
RHO GTPases ACTIVATE PKNs
Hit: 10/27 - FDR: 6.77E-1
RHO GTPases ACTIVATE PAKs
Hit: 10/24 - FDR: 5.06E-1
RHO GTPases ACTIVATE ROCKs
Hit: 12/23 - FDR: 1.49E-1
RHO GTPases ACTIVATE CIT
Hit: 15/36 - FDR: 2.76E-1
RHO GTPases ACTIVATE IQGAPs
Hit: 3/12 - FDR: 6.94E-1
RHO GTPases ACTIVATE KTN1
Hit: 5/10 - FDR: 6.4E-1
RHO GTPases ACTIVATE RHOTEKIN AND RHOPHILINs
NON-MUSCLE MYOSIN II DYNAMICS
VESICLE MOTILITY
NADPH oxidase complex
ACTIN FILAMENT POLYMERYSATION
GENE EXPRESSION
RHO GTPases ACTIVATE NADPH OXIDASES
Hit: 2/38 - FDR: 9.99E-1
Hit: 1/4 - FDR: 6.94E-1
RHO GTPases REGULATE CFTR TRAFFICKING
CFTR
CFTR TRAFFICKING TO PLASMA MEMBRANE
RHO GTPases ACTIVATE FORMINs
Hit: 87/149 - FDR: 1.84E-11
RHO GTPases ACTIVATE WASPs and WAVEs
Hit: 13/41 - FDR: 6.94E-1
2.08E1
1.12E0

B

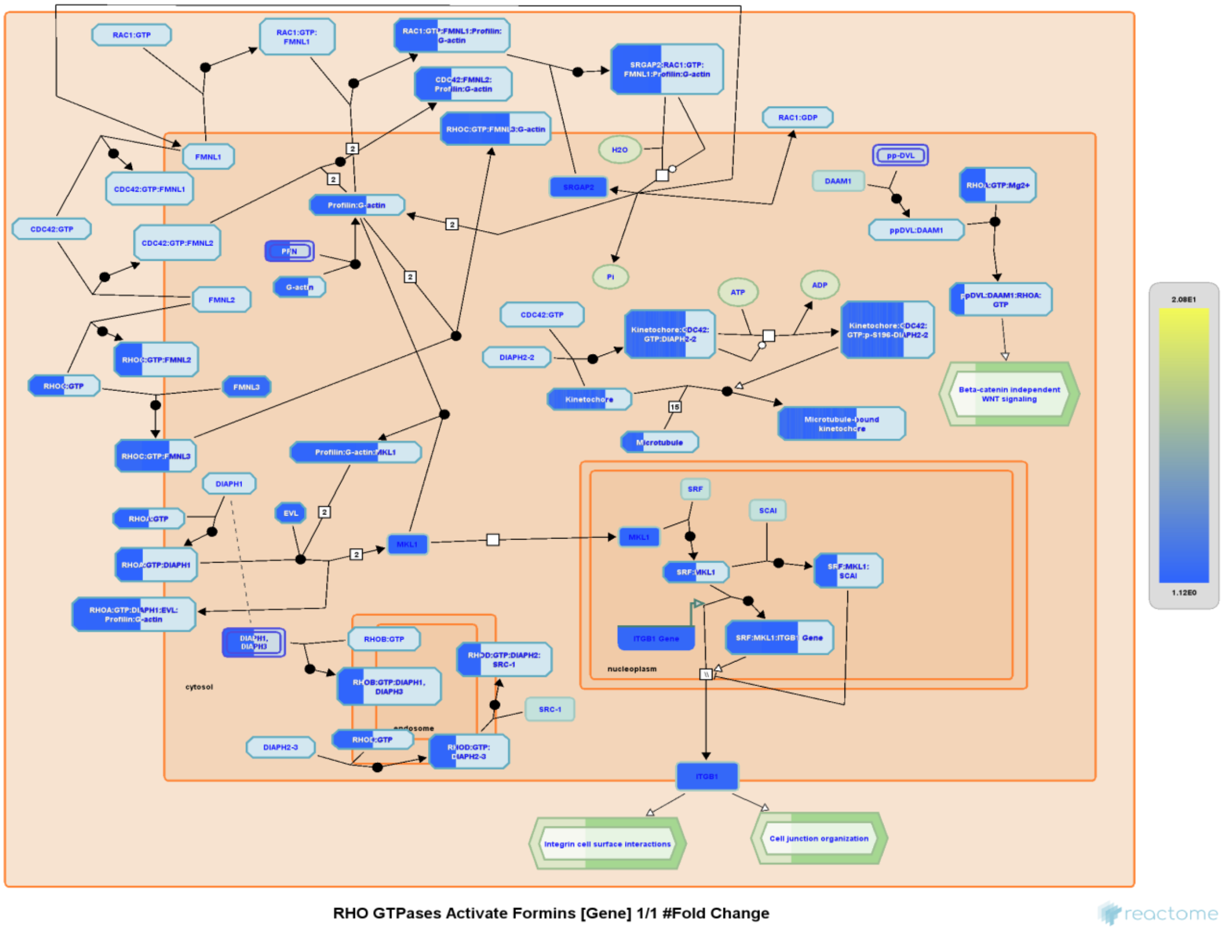
cytosol
nucleoplasm
endosome
Integrin cell surface interactions
Cell junction organization
Beta-catenin independent WNT signaling
2.08E1
1.12E0
RHO GTPases Activate Formins [Gene] 1/1 #Fold Change

C

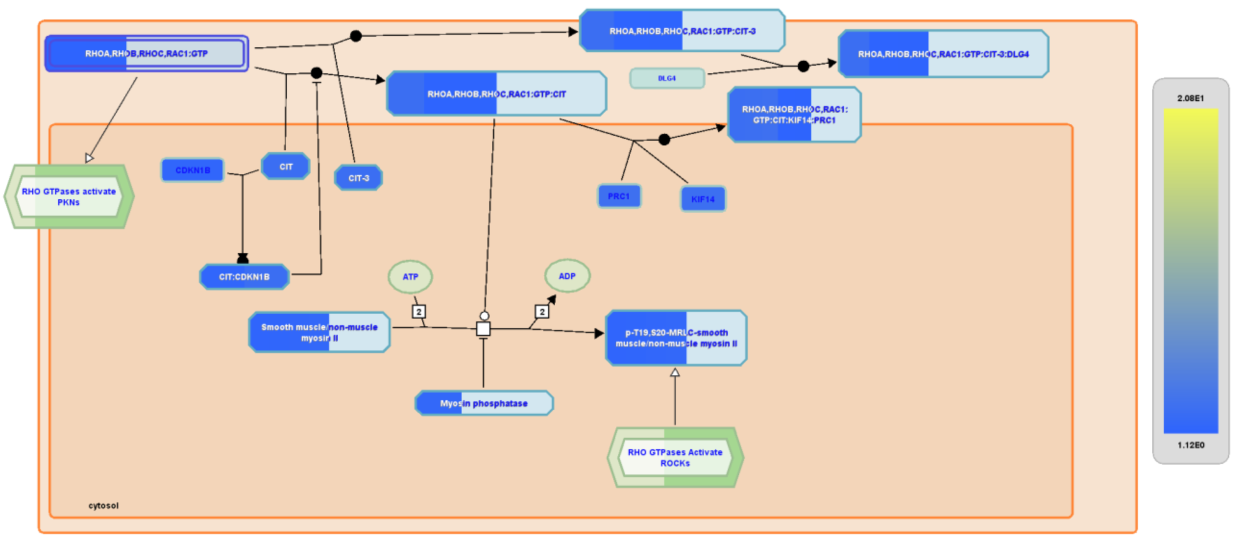

RHO GTPases activate CIT [Gene] 1/1 #Fold Change

D	Pathway name	#Entities found	#Entities total	Entities ratio	Entities pValue	Entities FDR
	RHO GTPase Effectors	137	326	0.022461	2.90E-10	3.42E-08
	Signaling by Rho GTPases	177	457	0.031487	3.80E-10	4.26E-08
	RHO GTPases Activate Formins	87	149	0.010266	1.03E-13	1.84E-11
	RHO GTPases activate CIT	12	23	0.001585	0.010101	0.148787
	Extracellular matrix organization	106	329	0.022668	0.00122	0.025628
	Degradation of the extracellular matrix	40	148	0.010197	0.219091	0.693597

E List of significantly decreased entities (i.e. genes) found within pathway:

RHO GTPase Effectors

CYFIP2;ERCC6L;GUCY1B1;ZWILCH;WIPF1;BUB1B;ACTB;ACTG1;CDC20;PPP1CC;TUBB6;KIF5B;CFL1;KNTC1;KPNA2;PDK1;GPT2;VASH2;CYBB;MRTFA;P3H4;KNL1;CDC25C;KIAA1217;SGO1;SGO2;MYL6;CLIP1;ZW10;PFN1;MYL9;HDGFL2;MAD1L1;NOTCH1;SEH1L;STXBP1;CDCA8;IQGAP3;PMF1;NUP160;MYL12A;SKA1;SKA2;DSN1;NUP85;UBR7;RAD21;RHPN1;SSR1;KIF3C;PATZ1;SRGAP2;KCNN4;PROM1;MYH10;PCK2;RCC2;PLK1;XRCC1;NDC80;ZWINT;KIF18A;DIAPH3;P4HA1;ARPC2;NF2;CALM2;ITGB1;FEN1;CDKN1B;ARPC1B;ARPC1A;KIF14;TUBA1B;TUBA1A;YWHAQ;NUF2;SPDL1;YWHAH;NUDC;H2AFZ;TUBB;H2AFV;PPP2R5D;CKAP5;RHOD;COMMD4;RHOA;CIT;TUBB2B;TUBB2A;KIF2A;INCENP;BIRC5;MYH9;KIF2C;EVL;KIFC3;PPP1R12B;DBN1;CIPC;MIS12;TYMS;CENPA;NSL1;AURKB;CENPC;PAK1;FLNA;MAPK1;NUP43;BUB3;BUB1;DYNC1I1;CENPT;CENPU;KDM4C;NDE1;F11;TUBB4B;ACVR2A;RNF145;CENPE;GALE;CENPF;ABI2;FAP;CENPH;PRC1;CENPI;DLC1;CYP20A1;CENPK;TACC3;CENPL;CENPM;CTNNB1;ESYT2;CENPN;B9D2;CENPO;CENPP;CENPQ;SPC24;SPC25;NUP37;MAD2L1;CENPS

Signaling by Rho GTPases

CYFIP2;ERCC6L;GUCY1B1;FAM13B;ZWILCH;WIPF1;NRM;BUB1B;FGF1;ACTB;NUBP1;ACTG1;EPS8;

CDC20;SYDE1;PPP1CC;TUBB6;KIF5B;ARHGDIA;CFL1;ARHGDIB;KNTC1;KPNA2;ARHGEF40;PDK1;GPT2;VASH2;CYBB;MRTFA;P3H4;KNL1;CDC25C;KIAA1217;SGO1;TIAM2;SGO2;MYL6;CLIP1;DEPDC1B;ZW10;PFN1;MYL9;HDGFL2;MAD1L1;NOTCH1;SEH1L;STXBP1;STK39;CDCA8;SLC41A1;PIK3R2;IQGAP3;PMF1;NUP160;MYL12A;SKA1;SKA2;ARHGAP22;DSN1;RACGAP1;NUP85;UBR7;RAD21;RHPN1;SSR1;KIF3C;PATZ1;SRGAP2;KCNN4;PROM1;ECT2;MYH10;PCK2;STARD13;BICRAL;RCC2;ARHGEF39;PLK1;XRCC1;NDC80;ZWINT;ARHGAP33;KIF18A;DIAPH3;P4HA1;ARPC2;DNAJC10;NF2;CALM2;RAD18;ITGB1;FEN1;CDKN1B;ARPC1B;RASGRF2;ARPC1A;KIF14;ARHGEF10L;PKD1;TUBA1B;TUBA1A;YWHAQ;NUF2;TIMP2;RAC3;SPDL1;YWHAH;PLEKHG3;ARHGEF10;NUDC;H2AFZ;TUBB;H2AFV;PPP2R5D;ELOVL7;RABGGTA;CKAP5;RHOD;COMMD4;NIPSNAP1;RHOA;CIT;GREM1;DEPDC7;TUBB2B;TUBB2A;KIF2A;INCENP;BIRC5;MYH9;KIF2C;EVL;ARHGEF2;KIFC3;RHOV;PPP1R12B;DBN1;CIPC;MIS12;ATP10A;TYMS;CENPA;NSL1;NAPG;AURKB;CENPC;PAK1;FLNA;MAPK1;NUP43;BUB3;BUB1;DYNC1I1;CENPT;CENPU;KDM4C;NDE1;F11;MYO9B;TUBB4B;ACVR2A;RNF145;CENPE;GALE;CENPF;ABI2;FAP;CENPH;PRC1;CENPI;DLC1;CYP20A1;CENPK;TACC3;CENPL;CENPM;CTNNB1;ESYT2;CENPN;B9D2;CENPO;CENPP;CENPQ;SPC24;SPC25;NUP37;MAD2L1;CENPS

RHO GTPases Activate Formins

ITGB1;ERCC6L;GUCY1B1;ZWILCH;BUB1B;ACTB;ACTG1;CDC20;PPP1CC;TUBB6;TUBA1B;TUBA1A;NUF2;KNTC1;SPDL1;NUDC;TUBB;VASH2;MRTFA;PPP2R5D;KNL1;RHOD;CKAP5;RHOA;KIAA1217;SGO1;SGO2;CLIP1;TUBB2B;TUBB2A;KIF2A;INCENP;ZW10;BIRC5;KIF2C;EVL;KIFC3;PFN1;MAD1L1;SEH1L;CIPC;STXBP1;MIS12;CDCA8;PMF1;CENPA;SKA1;NSL1;NUP160;AURKB;SKA2;CENPC;DSN1;NUP85;RAD21;SSR1;NUP43;BUB3;KIF3C;PATZ1;SRGAP2;BUB1;DYNC1I1;CENPT;CENPU;RCC2;NDE1;PLK1;F11;TUBB4B;NDC80;ZWINT;CENPE;GALE;KIF18A;CENPF;DIAPH3;CENPH;CENPI;DLC1;CENPK;TACC3;CENPL;CENPM;CENPN;B9D2;CENPO;CENPP;CENPQ;SPC24;SPC25;NUP37;MAD2L1;CENPS

RHO GTPases activate CIT

CDKN1B;STXBP1;KIF14;RHOA;CIT;MYL6;P4HA1;FAP;PRC1;MYH9;PPP1R12B;MYL9;MYH10

Extracellular matrix organization

SERPINE1;COL12A1;TNC;PLOD2;PLOD1;LOXL1;LOXL2;TBC1D31;RAVER2;BSG;ADPRHL1;ACTN1;P3H2;P3H1;ADAM19;ADAM17;LOX;COL4A2;COL4A1;ADAM12;COL8A2;ADAM9;ADAM8;VDAC1;PPIB;CD151;SDC3;HTRA1;WDR61;SPATA5;NID2;ACAT2;CHST12;ERI1;GRK6;SERPINH1;SRGAP2;CRTAP;TGFB1;JUP;NDC80;SLC16A13;COL1A1;FIBP;COL1A2;P4HA1;COL5A1;P4HA2;ITGA10;COL5A2;ITGA11;ITGB1;PTPRS;ITGB5;SLC44A2;RASGRF2;SLC2A1;LAMC2;MCM10;ING1;ADAMTS2;TIMP2;MAN1A1;COLGALT1;GPX1;MME;MMD;MMP1;ITGA3;CEP295;TEX10;MMP2;ITGA1;MASTL;CKAP4;ASPN;MMP11;MMP14;COL2A1;COL6A2;MMP17;PXDN;PECAM1;ITGA8;ITGA7;MYH9;ITGA5;CRELD2;METTL26;PLEC;TMPRSS6;LAMA3;LRP4;THBS1;ACAN;CYTH2;FIP1L1;TMEM204;SPP1;LRRC8D;NCAM1;JAM2;MDH2;LUM;F11;ADAT2;NEDD1;RNF145;SH3D21;CAPN10;P4HB;B9D2

Degradation of the extracellular matrix

TMPRSS6;SLC44A2;LAMA3;COL12A1;HTRA1;TNC;LAMC2;WDR61;ACAN;FIP1L1;BSG;TIMP2;SPP1;MAN1A1;MME;MMP1;MDH2;MMP2;TEX10;F11;NDC80;COL1A1;RNF145;MMP11;MMP14;ADAM17;COL2A1;COL1A2;COL5A1;COL4A2;COL4A1;CAPN10;COL6A2;COL5A2;MMP17;COL8A2;ADAM9;MYH9;ADAM8

Fig. S6: Pathways with significantly (padj<0.05) increased target gene expression in CN03+TNFα vs. TNFα groups. Reactome pathway for (A) Rho GTPases effectors; (B) Rho GTPases FORMIN pathway; (C) Rho GTPases CIT pathway. Color bar indicates fold change in CN03+TNFα vs. TNFα. (D) Quantitative statistics from Reactome

pathway analysis and (E) list of significant entities (i.e. genes) found within each pathway.

A. NF-kb related pathways whose target genes significantly (padjs<0.05) increased in TNFα vs untreated

Pathway name	#Entities found	#Entities total	Entities ratio	Entities pValue	Entities FDR
NIK-->noncanonical NF-kB signaling	44	61	0.004203	1.49E-06	2.38E-04
Interleukin-1 family signaling	89	163	0.011231	2.51E-06	2.79E-04
TNFR2 non-canonical NF-kB pathway	63	104	0.007165	3.28E-06	3.48E-04
Dectin-1 mediated noncanonical NF-kB signaling	45	66	0.004547	4.73E-06	4.35E-04
Activation of NF-kappaB in B cells	47	72	0.004961	8.88E-06	6.30E-04

B. List of significantly increased entities (i.e. genes) found within pathways listed in table above (a):

NIK-->noncanonical NF-kB signaling

THRB;PLAT;PSMD8;RABGEF1;PSMD7;PSMD5;PSMD2;PSMD3;PSMD1;TPRA1;FBXW11;TAP2;TAP1;PSMA5;PSMA6;PSMA3;PSMA4;PSMA1;PSMA2;PSME3;PSME1;PSME2;PSMD12;PSMD11;MTFP1;PSMD14;CUL1;LRP8;PSMB10;PSMA7;RELB;PSMB6;PSMB7;PSMB4;PSMB5;UBB;PSMB2;ZNHIT3;PSMF1;USP25;PSMB8;NFKB2;PSMB9;PSMC5;PSMC4;REL;MAP3K10;TUBGCP4;MAP3K14;UBE2M

Interleukin-1 family signaling

IL1RN;THRB;UBE2D1;PTPN22;PLAT;IL1RAP;ACTB;IKBKB;PSMD8;RABGEF1;IL18RAP;IL36A;TBK1;PSMD7;IL36B;PSMD5;PSMD2;PSMD3;PSMD1;MAP3K8;MAP2K4;MAP2K1;TPRA1;H2AFY;USP5;FBXW11;RIPK2;TAP2;IL18;TAP1;IRAK3;PSMA5;QTRT2;PSMA6;PSMA3;PSMA4;PSMA1;PSMA2;PSME3;PSME1;PELI1;PSME2;SQSTM1;PSMD12;PSMD11;PSMD14;CUL1;NOD2;LRP8;TOB1;RELA;PSMB10;ACAT2;PSMA7;PSMB6;PSMB7;IL1RL1;PSMB4;PSMB5;UBB;IRAK1;PSMB2;ZNHIT3;IRAK2;PSMF1;USP25;MAP3K3;PTPN18;PTPN11;PTPN12;HPS5;PTPN13;NFKB1;PSMB8;KTN1;NFKB2;PSMB9;NFKBIA;PSMC5;PSMC4;TNIP2;REL;MDM2;UBE2N;PTPN9;MAP3K10;TAB2;TUBGCP4;PTPN4;PTPN2;IL18R1;NFKBIB

TNFR2 non-canonical NF-kB pathway

CD40;TNFRSF6B;THRB;PLAT;TNFSF13B;PSMD8;RABGEF1;PSMD7;PSMD5;PSMD2;PSMD3;TNFRSF8;PSMD1;CAP1;TPRA1;ZGPAT;TNFRSF12A;FBXW11;TNFRSF18;TAP2;TAP1;TRAF2;VRK1;TNFRSF1A;PSMA5;PSMA6;PSMA3;PSMA4;PSMA1;TRAF3;PSMA2;PSME3;PSME1;PSME2;BIRC2;BIRC3;PSMD12;PSMD11;MTFP1;PSMD14;CUL1;TNFRSF11B;TNFRSF11A;LRP8;PSMB10;PSMA7;RELB;RAB21;PSMB6;PSMB7;PSMB4;PSMB5;UBB;PSMB2;ZNHIT3;TRPM7;PSMF1;TNFSF18;USP25;TNFSF15;TNFRSF9;PSMB8;NFKB2;PSMB9;PSMC5;PSMC4;TNFSF4;REL;MAP3K10;LTBR;TUBGCP4;MAP3K14;UBE2M

Dectin-1 mediated noncanonical NF-kB signaling45

THRB;PLAT;PSMD8;RABGEF1;PSMD7;PSMD5;PSMD2;PSMD3;PSMD1;TPRA1;FBXW11;TAP2;TAP1;PSMA5;PSMA6;PSMA3;PSMA4;PSMA1;PSMA2;PSME3;PSME1;PSME2;PSMD12;PSMD11;MTFP1;PSMD14;CUL1;LRP8;RELA;PSMB10;PSMA7;RELB;PSMB6;PSMB7;PSMB4;PSMB5;UBB;PSMB2;ZNHIT3;PSMF1;USP25;PSMB8;NFKB2;PSMB9;PSMC5;PSMC4;REL;MAP3K10;TUBGCP4;MAP3K14;UBE2M

Activation of NF-kappaB in B cells

THRB;PLAT;IKBKB;PSMD8;RABGEF1;PSMD7;PSMD5;PSMD2;PSMD3;PSMD1;TPRA1;FBXW11;TAP2;TAP1;PSMA5;PSMA6;PSMA3;PSMA4;PSMA1;PSMA2;PSME3;PSME1;PSME2;PSMD12;PSMD11;PSMD14;CUL1;LRP8;MALT1;RELA;PSMB10;PSMA7;PSMB6;PSMB7;PSMB4;PSMB5;UBB;PSMB2;ZNHIT3;PSMF1;USP25;NFKB1;PSMB8;PSMB9;NFKBIA;PSMC5;PSMC4;REL;MAP3K10;TUBGCP4;NFKBIE;NFKBIB

C. NF-kb related pathways whose target genes significantly (padjs<0.05) decreased in CN03+TNFα vs TNFα

Pathway name	#Entities found	#Entities total	Entities ratio	Entities pValue	Entities FDR
Interleukin-1 signaling	37	109	0.00751	0.005961	0.662365
NF-kB activation through FADD/RIP-1 pathway mediated by caspase-8 and -10	8	13	8.96E-04	0.008201	0.662365
NIK-->noncanonical NF-kB signaling	22	61	0.004203	0.01581	0.662365
Activation of NF-kappaB in B cells	24	72	0.004961	0.027443	0.662365
Dectin-1 mediated noncanonical NF-kB signaling	22	66	0.004547	0.033516	0.662365

D. List of significantly decreased entities (i.e. genes) found within pathways listed in table above (c):

Interleukin-1 signaling

APP;PSMD12;CUL1;UBE2D1;IL1RAP;NOD2;TOB1;PSMB10;EGFR;IKBKB;PSMB4;PSMB2;NKIRAS2;UBC;MAP3K8;PSMF1;USP25;FBXW11;SORT1;RIPK2;IL1R1;TAP1;IRAK3;IRAK4;PSMB8;PSMB9;NFKBIA;PSMA6;PSMA3;SGSM2;PSME4;EEF1A2;REL;PSME1;PSME2;TAB2;TUBGCP4;SQSTM1;MYD88;NFKBIB

NF-kB activation through FADD/RIP-1 pathway mediated by caspase-8 and -10

IFIH1;IKBKB;MAVS;CASP8;DDX58;TRIM25;RIPK1;CFLAR

NIK-->noncanonical NF-kB signaling

USP25;PSMD12;FBXW11;SORT1;CUL1;TAP1;PSMB10;PSMB8;RELB;PSMB9;PSMA6;PSMB4;PSMA3;PSMB2;UBC;SGSM2;PSME4;EEF1A2;REL;PSME1;PSME2;TUBGCP4;PSMF1;MAP3K14

Activation of NF-kappaB in B cells

PSMD12;CUL1;PSMB10;IKBKB;PSMB4;PSMB2;UBC;PSMF1;USP25;FBXW11;SORT1;TAP1;PSMB8;PSMB9;NFKBIA;PSMA6;PSMA3;SGSM2;PSME4;EEF1A2;REL;PSME1;PSME2;TUBGCP4;NFKBIE;NFKBIB

Dectin-1 mediated noncanonical NF-kB signaling

USP25;PSMD12;FBXW11;SORT1;CUL1;TAP1;PSMB10;PSMB8;RELB;PSMB9;PSMA6;PSMB4;PSMA3;PSMB2;UBC;SGSM2;PSME4;EEF1A2;REL;PSME1;PSME2;TUBGCP4;PSMF1;MAP3K14

TNFR2 non-canonical NF-kB pathway

PSMD12;TNFRSF6B;CUL1;TNFRSF11B;PSMB10;RELB;TNFSF13B;PSMB4;PSMB2;UBC;PSMF1;USP25;FBXW11;SORT1;TNFRSF19;TAP1;TRAF2;PSMB8;TNFRSF1A;PSMB9;PSMA6;PSMA3;RASA1;SGSM2;PSME4;EEF1A2;REL;PSME1;PSME2;TUBGCP4;MAP3K14;BIRC3

Fig. S7: NF-κB pathways with significant (padj<0.05) changes in gene expression. (A) Quantitative statistics from Reactome pathway analysis and (B) list of significant entities (i.e. genes) found within each pathway in TNFα vs. untreated groups. (C) Quantitative statistics from Reactome pathway analysis and (D) list of significant entities (i.e. genes) found within each pathway in CN03+TNFα vs. TNFα groups.

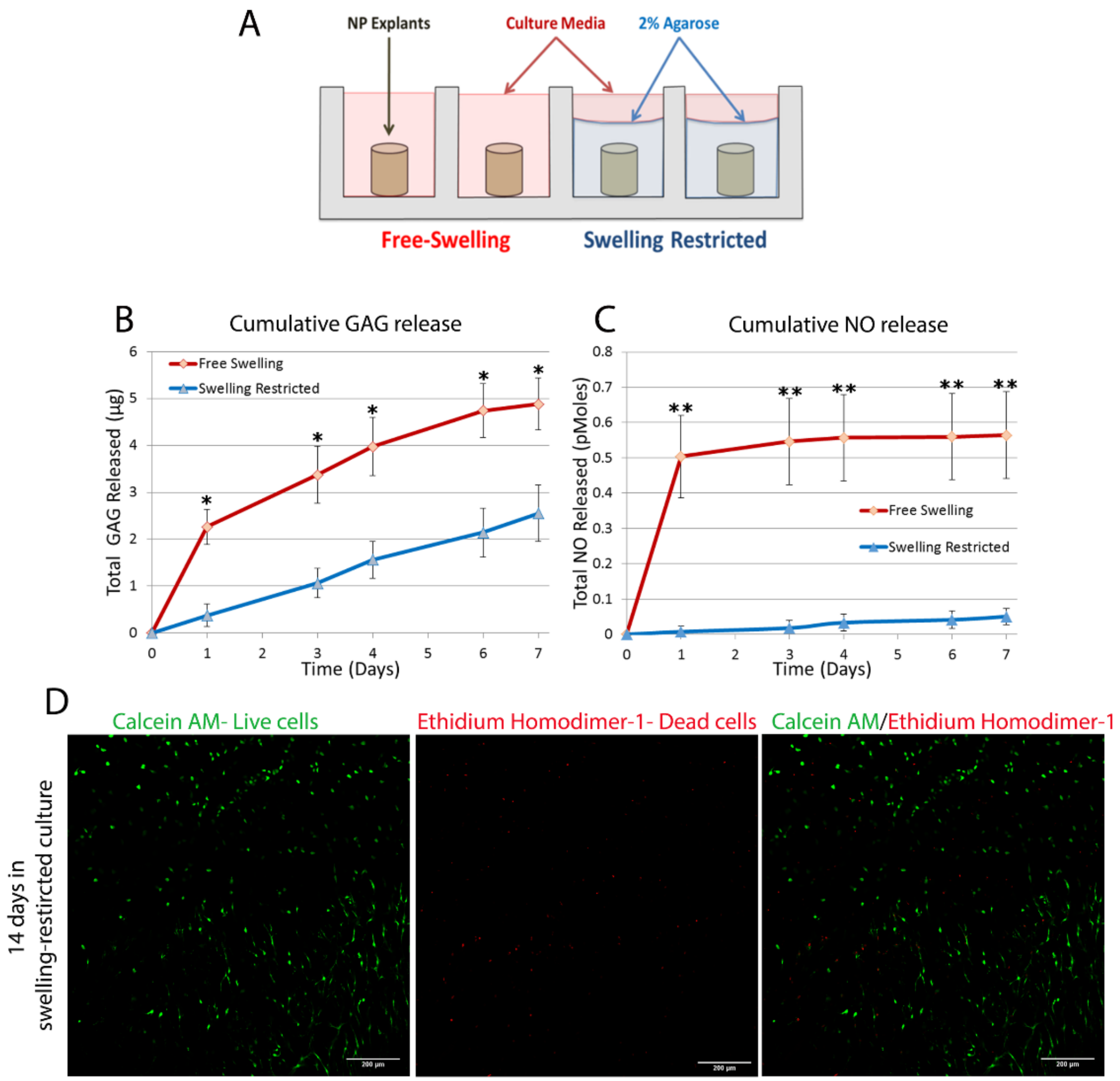

Fig. S8: Swelling restricted explant-in-hydrogel organ culture system preserves NP explant integrity *in vitro*. (**A**) Schematic of explant-in-hydrogel system that restrict explant swelling *in vitro*. NP explants were cultured either cast in agarose hydrogel to prevent swelling (blue) or in free swelling conditions (red). (**B**) Swelling restricted explants showed reduced cumulative GAG release into the media (N_{Unt}=3, $N_{TNF\alpha}$=3, *p<0.01 vs. swelling restricted) at all time points. (**C**) Nitric oxide (NO) release into the media had a burst release initially in free swelling culture. Swelling restricted culture reduced NO release into the media at all time points compared to free swelling (N_{Unt}=6, $N_{TNF\alpha}$=6, **$p<10^{-7}$ vs. explant-in-hydrogel system). Subpanel b and c: mean ± SD. (**D**) Explants in swelling-restricted culture (2% agarose) showing live cells (Calcein AM, green) and dead cells (Ethidium Homodimer-1, red) after 14 days in culture. Scale bar is 200 µm.
